# CDK4/6 Inhibitor Priming Enhances PD‐1 Blockade via Sell^hi^ Neutrophil‐Induced Stat5a^+^ Progenitor Exhausted CD8^+^ T Cell

**DOI:** 10.1002/advs.202510501

**Published:** 2025-09-11

**Authors:** Yu Zhang, Bao Sun, Rong Zhou, Zizhen Gong, Yong Han, Wenjie Tao, Chaoji Shi, Wuchang Zhang, Laikui Liu, Zhiyuan Zhang, Jiang Li, Qi Zhu, Weiwen Zhu, Ziyue Gu

**Affiliations:** ^1^ Department of Oral Maxillofacial and Head and Neck Oncology Shanghai Ninth People's Hospital Shanghai Jiao Tong University School of Medicine Shanghai 200011 China; ^2^ College of Stomatology Shanghai Jiao Tong University Shanghai 200011 China; ^3^ National Center for Stomatology Shanghai 200011 China; ^4^ National Clinical Research Center for Oral Diseases Shanghai 200011 China; ^5^ Shanghai Key Laboratory of Stomatology Shanghai 200011 China; ^6^ Department of Oral Mucosal Diseases The Affiliated Stomatological Hospital of Nanjing Medical University Nanjing 210029 China; ^7^ State Key Laboratory Cultivation Base of Research Prevention and Treatment for Oral Diseases Nanjing Medical University Nanjing 210029 China; ^8^ Jiangsu Province Engineering Research Center of Stomatological Translational Medicine Nanjing Medical University Nanjing 210029 China; ^9^ Department of Oral Pathology Shanghai Ninth People's Hospital Shanghai Jiao Tong University School of Medicine Shanghai 200011 China; ^10^ Laboratory of Oral Microbiota and Systemic Diseases Shanghai Ninth People's Hospital College of Stomatology Shanghai Jiao Tong University School of Medicine Shanghai 200011 China; ^11^ Laboratory of Molecular Signaling Division of Oral Biology and Medicine School of Dentistry University of California Los Angeles Los Angeles CA 90095 USA; ^12^ Collaborative Innovation Center for Cancer Personalized Medicine Nanjing Medical University Nanjing 210029 China

**Keywords:** HNSCC, Sell(hi) neutrophils, Stat5a, CD8+Tpex, CDK4/6i priming

## Abstract

Cell cycle pathway, especially via cyclin D1‐CDK4/6 signaling, is enriched in immunotherapy‐resistant and immune‐excluded tumors. CDK4/6 inhibitor (CDK4/6i) induces antitumor immune phenotypes by targeting both tumor and immune cells, enhancing immune checkpoint blockade (ICB), but optimal combination modalities and the corresponding cellular mechanisms remain unclear. Here, it is shown that activation of tumor cell‐intrinsic cyclin D1‐CDK4/6 signaling is associated with low tumor‐infiltrating lymphocyte populations and immunotherapy resistance in head and neck squamous cell carcinoma (HNSCC). Comparison of sequential versus combinatorial regimens in subcutaneous or orthotopic HNSCC mice revealed that CDK4/6i priming before anti‐PD‐1 enhances response durability by promoting CD8^+^ and CD4^+^ T cell infiltration and decreasing overall neutrophil abundance. Mechanistically, IL15‐secreted Sell(hi) neutrophils induced Stat5a+ progenitor exhausted CD8^+^ T cells contributed to the antitumor effect of CDK4/6i priming modalities. Together with corroborating evidence from a clinically relevant patient‐derived‐organoid‐TIL (PDO‐TIL) co‐culture model, these findings support further clinical testing of brief CDK4/6i dosing before anti‐PD‐1 to improve ICB efficacy.

## Graphical abstract

Activated tumor‐intrinsic cyclin D1‐CDK4/6 signaling attenuates lymphocyte infiltration, and consequently, and immunotherapy resistance in HNSCC. It is discovered that brief CDK4/6i priming before anti‐PD‐1 results in enhanced durability of ICB response durability through Stat5a^+^ progenitor exhausted CD8^+^ T cells induced by IL15‐secreted Sell^(hi)^ neutrophils.

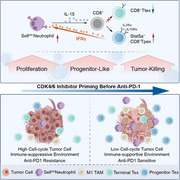

## 1 Introduction

Programmed cell death protein‐1 (PD‐1) related therapies have revolutionized cancer treatment. However, poor responses to immune checkpoint blockade (ICB) and resistance remain substantial challenges.^[^ ^1^ ^]^ Genetic alterations and oncogenic pathways intrinsic to cancer can elicit a cold tumor microenvironment (TME).^[^ ^2^ ^]^ Cell cycle pathway activation contributes to the immunosuppressive TME and resistance.^[^ ^3^, ^4^ ^]^ Deregulation of cell‐cycle signaling, mediated by CCND1 and CDKN2A aberrations, is in 50%+ of HPV‐negative head and neck squamous cell carcinomas (HNSCC).^[^ ^5^ ^]^ The role of aberrant cell‐cycle signaling in the immunosuppressive TME of HNSCC and whether blocking cyclin D‐CDK4/6 signaling could overcome immunotherapy resistance remains unclear.

PD‐1 ICB can reinvigorate exhausted CD8^+^ T‐cells (Tex), enhance their effector function, and improve tumor control, but most patients do not achieve a durable clinical response.^[^ ^6^ ^]^ T‐cell exhaustion is a progressive developmental process, and Tex are heterogeneous, including PD‐1+TCF1+TIM‐3‐ progenitor Tex (Tpex) and PD‐1+TCF1‐TIM‐3+ terminal Tex (Ttex).^[^ ^7^ ^]^ The Tpex population exhibits greater plasticity, retains its proliferative and self‐renewal capacity, and expands after ICB, giving rise to terminally exhausted cells.^[^ ^8^ ^]^ Ttex cells express high levels of PD‐1 and co‐express other inhibitory receptors (TIM‐3, LAG3, TIGIT), retain some cytotoxic potential, but are non‐plastic following ICB.^[^ ^9^ ^]^ Developing strategies to revert Ttex or maintain progenitor Tex during ICB therapy is crucial.

CDK4/6 inhibitors (CDK4/6i), targeting cyclin D‐CDK4/6, are approved for treating hormone receptor–positive breast cancer.^[^ ^10^ ^]^ Beyond inducing tumor cell cytostasis, CDK4/6i‐mediated inflammation in the tumor microenvironment enhances antigen presentation on tumor cells, increases interferon production, and promotes T cell infiltration into tumors.^[^ ^11^ ^]^ CDK4/6 inhibitors also have immunomodulatory effects, skewing T cells toward a state of self‐renewal and multiple differentiation potential.^[^ ^12^, ^13^ ^]^ Consequently, combination therapies of CDK4/6 inhibitors with ICB show promising outcomes,^[^ ^14^, ^15^ ^]^ with several clinical trials (NCT02778685, NCT04438824, NCT04360941, and NCT03498378). However, the lack of breakthroughs is likely attributable to two factors. First, high‐grade toxicities often encountered with combined CDK4/6i and ICB treatment have made it difficult to assess long‐term clinical outcomes.^[^ ^16^ ^]^ Second, the persistent anti‐proliferative effect on T cells exerted by continuous CDK4/6i dosing might limit the expansion of cytotoxic T cells following ICB.^[^ ^13^ ^]^

Notably, concurrent application of CDK4/6i and ICB does not yield tumor regression and showed limited anti‐tumor effect in preclinical models.^[^ ^17^, ^18^ ^]^ Tumor regression is observed only with sequential treatment, where CDK4/6i abemaciclib is administered first, followed by ICB. This approach results in long‐term anti‐tumor immune memory in the mice.^[^ ^15^ ^]^ Conversely, in a melanoma syngeneic model, combining anti‐CTLA‐4 with anti‐PD‐1 showed a strong synergistic anti‐tumor effect when CDK4/6i abemaciclib was administered sequentially after the immune checkpoint inhibitor.^[^ ^3^ ^]^ Both studies suggest that sequential therapy might maximize the synergistic effect between CDK4/6i and ICB. However, mechanisms underlying the improved antitumor capacity of sequential therapy compared to anti‐PD‐1 monotherapy remain unclear. This hinders clinical application of the CDK4/6i‐plus‐anti‐PD‐1 combination in solid tumors.

Here, we investigated the effects of cell cycle pathway activation on the TME and ICB response and elucidated mechanisms underlying the optimized sequential combination of CDK4/6i and ICB using syngeneic tumor models. We confirmed that activation of cyclin D‐CDK4/6 signaling impedes lymphocyte infiltration and impairs immunotherapy response in HNSCC. Notably, we demonstrated that priming with CDK4/6i before anti‐PD‐1 therapy elicited superior antitumor immunity compared to single‐agent or other combination treatments. This was achieved by promoting the infiltration of pro‐inflammatory M1‐like macrophages, effector memory CD4^+^ and CD8^+^ T cells, and reducing overall neutrophil levels. Strikingly, we observed that Sell(hi) neutrophils mediated the induction of Stat5a+ Tpex phenotype through the IL15‐Stat5a axis in the CDK4/6i priming groups. Together, our results provide a comprehensive mechanistic insight into the promising clinical modality of CDK4/6i priming prior to ICB therapy.

## 2 Results

### 2.1 Cyclin D1‐CDK4/6 Signaling Modulates Lymphocyte Infiltration and ICB Resistance

**Figure 1:**
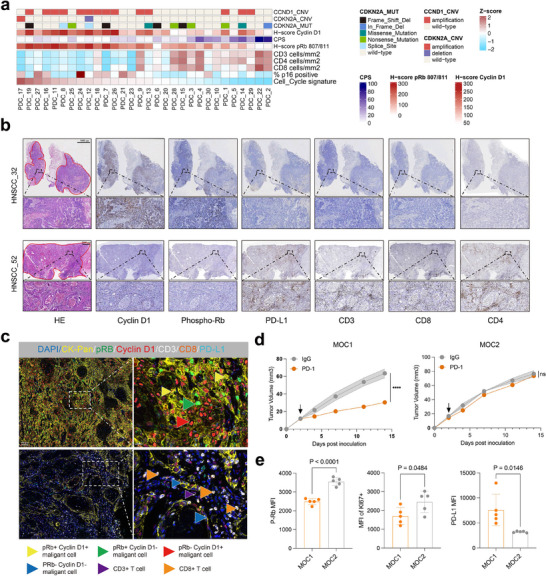
Cyclin D1‐CDK4/6 signaling is associated with T cell exclusion and immunotherapy resistance. a) Heatmap comparing cell cycle signature, H‐score of Rb phosphorylation, Cyclin D1, counts of CD3^+^, CD4^+^, CD8^+^ T cells, p16 positive percentage and the combined positive score of PD‐L1 (CPS), with genomic aberrations annotated. Cell cycle signature and genomic aberrations of 30 PDC models were annotated using RNA‐sequencing and Whole exome sequencing respectively. b) Representative images of IHC staining of Cyclin D1, Phospho‐Rb, PD‐L1, CD3, CD4, CD8 in cell cycle signature high and low HNSCC patients. Scale bar, 100, 20 µm. c) Representative images of mIF showing cell cycle signature high HNSCC patient coupling with high expression of Cyclin D1, Phospho‐Rb and low PD‐L1 expression and CD3, CD8 infiltration, and cell cycle signature low HNSCC patient coupling with low expression of Cyclin D1, Phospho‐Rb and high PD‐L1 expression and CD3, CD8 infiltration. Scale bar, 100, 20 µm. d) Mean tumor growth curves showing tumor volume in MOC1 and MOC2 bearing‐mice treated with IgG or anti‐PD‐1 antibodies. Data are mean ± standard error of mean (s.e.m) (n = 5–6 mice per group). Two‐way ANOVA analysis. e) Flow cytometry of Phospho‐Rb, Ki67, PD‐L1 expressed in Epcam^+^ tumor cell in MOC1 or MOC2 tumors (n = 5). MFI, mean fluorescence intensity. Data are presented as mean ± standard deviation (s.d). Statistical significance was determined by two‐sided unpaired t‐test.

As tumor intrinsic cell cycling drives T cell exclusion and resistance to checkpoint blockade,^[^ ^3^, ^19^ ^]^ we examined prognosis in The Cancer Genome Atlas (TCGA)‐head and neck squamous cell carcinoma (HNSCC) dataset and an immunotherapy‐related cohort (GSE159067). This analysis revealed that high expression of cell cycle signature, which was defined as a program related to immune evasion,^[^ ^3^ ^]^ was associated with dismal prognosis in the immunotherapy cohort, but no such prognostically informative relationship was found in TCGA (Figure S1a, Supporting Information), suggesting cell cycle signature was specifically related to immunotherapy outcomes in HNSCC (Figure S1a, Supporting Information). Cell cycle‐associated pathways were shown to have a prominent role in HPV‐negative HNSCC, especially for cyclin D1‐CDK4/6 signaling. We conducted whole exome sequencing (WES) and RNA sequencing (RNA‐seq) in 30 in‐house HNSCC PDCs (patient derived cell lines)^[^ ^20^ ^]^ to screen for causal mutations/deletions/amplifications in CDKN2A and/or CCND1 and score cell cycle gene signature (**Figure** **1**a). Additionally, histochemistry scores (H‐scores) obtained by staining for cyclin D1‐CDK4/6 marker proteins, Cyclin D1 and Phospho‐Rb in paired tumors (Figure 1a,b; Table S1, Supporting Information) showed that patients harboring CCND1 amplification had increased CCND1 protein levels, elevated downstream Rb phosphorylation, and generally upregulated cell cycle gene signature, which collectively reflected cyclin D1‐CDK4/6 pathway activation (Figure 1a; Figure S1b, Supporting Information). Multiplex immunohistochemistry staining also showed that patients with low cell cycle signature tend to accumulate more CD3 and CD8 T cells in tumor and vice versa (Figure 1c; Figure S1c, Supporting Information, *P* = 0004 and *P* = 0014). To investigate whether tumor intrinsic cyclin D1‐CDK4/6 signaling was linked to response to immunotherapy, we utilized two HNSCC murine models that exhibit sensitivity (MOC1) or resistance (MOC2) to PD‐1 blockade in vivo (Figure 1d; Figure S1d, Supporting Information). Flow cytometry analysis indicated that cycling tumor cell (Ki67^hi^ and pRb^hi^ Epcam^+^ cells) abundance was significantly higher in MOC2 tumors compared with sensitive MOC1 tumor (Figure 1e). Surprisingly, CD4^+^ and CD8^+^ T cell, but not NK, infiltration and proliferation were significantly decreased in resistant MOC2 tumors compared to that in MOC1 (Figure S1e,f, Supporting Information), indicating a more immunosuppressive microenvironment in cycling (i.e., resistant) tumors. Collectively, these results suggested that cell cycle signature, especially cyclin D1‐CDK4/6, likely contributes to lymphocyte infiltration and immunotherapy response in HNSCC.

### 2.2 CDK4/6i Priming Preceding Anti‐PD‐1 Maximizes Antitumor Efficacy

**Figure 2:**
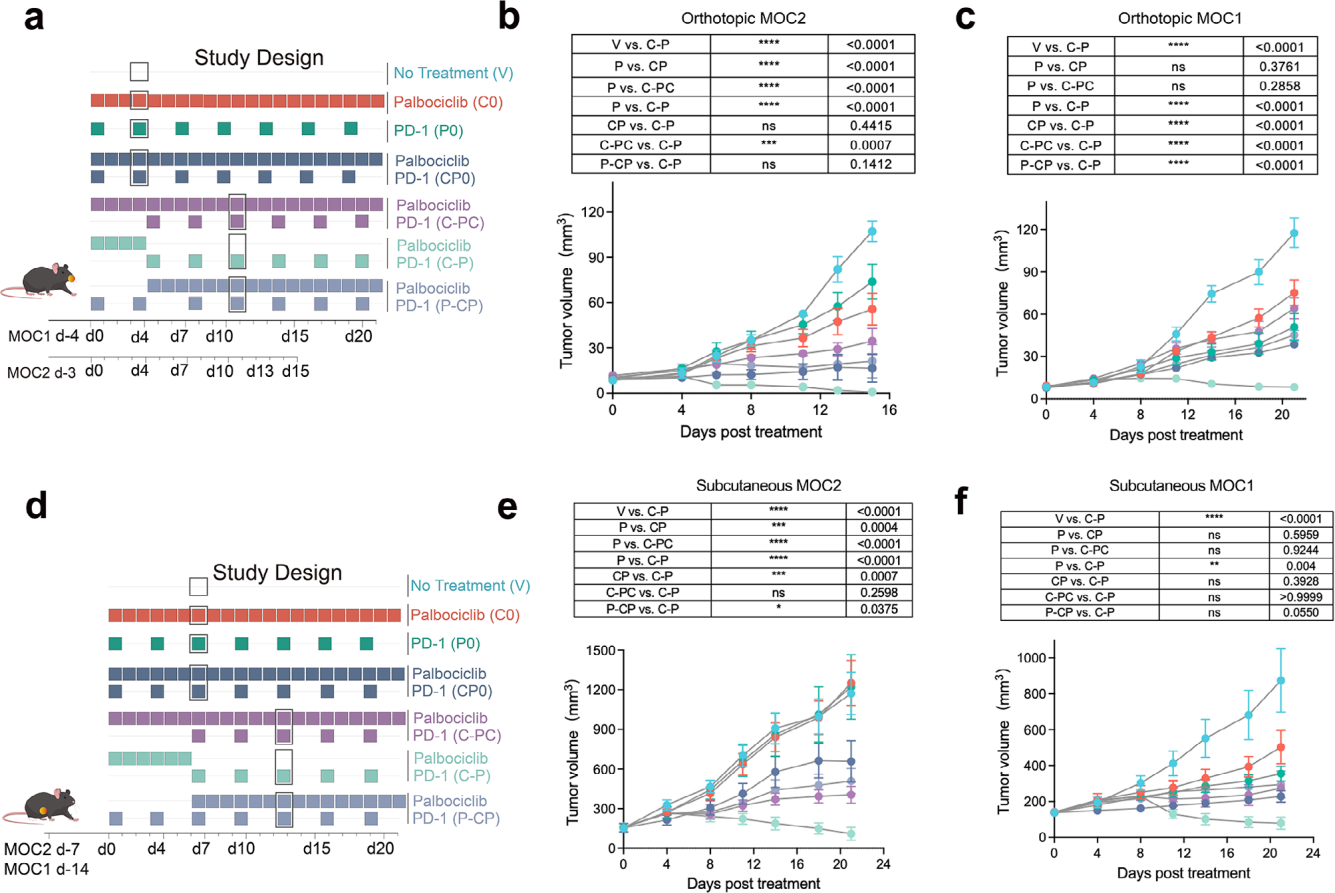
CDK4/6i priming before PD‐1 blockade shows superior antitumor activity than concurrent regimens and overcome immunotherapy resistance. a–d) Preclinical treatment regimen with anti‐PD‐1 day0‐7, CDK4/6i day0‐7, concurrent treatment of anti‐PD‐1 and CDK4/6i day0, palbociclib priming 7 days before anti‐PD‐1 combination, CDK4/6i priming 7 days before anti‐PD‐1 monotherapy and anti‐PD‐1 priming 7 days before CDK4/6i combination cisplatin for mice bearing orthotopic and subcutaneous MOC1 and MOC2. For the CDK4/6i priming strategy, CDK4/6i was administered from day 0 (randomization time) to day 4 for orthotopic and from day 0 (randomization time) to day 7 for subcutaneous tumor after tumor inoculation. CDK4/6i priming was initiated on day 3–4 after orthotopic tumor inoculation. For subcutaneous tumors, it was initiated when the tumor volumes reached 100–150 mm^3^. Dark gray circles indicate regimen and time points for CyTOF analysis in Figure 3. Tongue tumor tissues were collected from three syngeneic subcutaneous or orthotopic tumor models at time points and in treatment regimens including: 1) vehicle, 2) CDK4/6i, 3) anti‐PD‐1, 4) CDK4/6i + anti‐PD‐1 (on d4 for orthotopic and d7 for subcutaneous tumor); 5) C‐PC, 6) C‐P, 7) P‐CP (on d11 for orthotopic and d13 for subcutaneous tumor) for CyTOF analysis. Mouse tumor growth curves in response to treatment strategy outlined in (a), n = 5‐6, two‐way ANOVA test. Error bands were shaded. e,f) Mouse tumor growth curves in response to treatment strategy outlined in (d), n = 5, two‐way ANOVA test. Error bands were shaded. Data in (b, c, e, f) are presented as mean ± S.E.M.

While CDK4/6i plus anti‐PD‐1 and similar combination therapies have shown promise in both preclinical^[^ ^15^, ^18^, ^21^ ^]^ and clinical trials,^[^ ^22^, ^23^ ^]^ the anti‐tumor efficacy of sequential targeted therapy followed by immunotherapy has been largely overlooked. To explore possible benefits of sequential therapies, we used syngeneic subcutaneous and orthotopic HNSCC tumor model mice to test whether brief CDK4/6i pretreatment could improve subsequent response to anti‐PD‐1, with or without continued CDK4/6i dosing. Defining Day 0 (d0) as the time average tumor volume reaches 100–150 mm^3^ for subcutaneous models and 3–4 days after tumor inoculation for orthotopic models and d0‐d7 as the CDK4/6i priming period (**Figure** **2**a–d), mice with orthotopic or subcutaneous (sensitive) MOC1 or (resistant) MOC2 implant were treated with no active treatment, single‐agent treatment, simultaneous or sequential combination treatments, including: 1) vehicle (V, starting on d7); 2) CDK4/6i (C0, starting on d0); 3) anti‐PD‐1 (P0, starting on d0); 4) CDK4/6i + anti‐PD‐1 (CP0, concurrently starting on d0); and 5) sequential combinations (C‐PC, CDK4/6i followed by CDK4/6i + anti‐PD‐1; C‐P, CDK4/6i followed by anti‐PD‐1; or P‐CP, anti‐PD‐1 followed by CDK4/6i + anti‐PD‐1) (Figure 2a–d). Tests of the CDK4/6i priming effect showed that the C‐P regimen had stronger anti‐tumor effects than C‐PC in both models, consistently resulting in the most extensive and durable tumor regression (Figure 2b,c,e,f). In the MOC2 model, C0 and P0 showed minimal (orthotopic) to no inhibitory effects on tumor growth (subcutaneous), while the CP0 regimen could significantly inhibit tumor volume (Figure 2b,c; Figure S2a–c, Supporting Information). In the MOC1 model, C0/P0 could effectively delay tumor growth, and the CP0 regimen elicited a slight synergistic antitumor activity in both subcutaneous and orthotopic tumor models (Figure 2e,f; Figure S2b–d, Supporting Information). Notably, P‐CP regimen showed no obvious difference in anti‐tumor effects from that of CP0 in any of the four models (Figure 2b,c,e,f). Further, mice in the C‐P group also showed body weight gain when compared to original body weight (although less than 10%), suggesting an acceptable drug administration strategy (Figure S2e, Supporting Information). These results indicated that CDK4/6i priming followed by anti‐PD‐1 alone conferred the most durable antitumor activity, regardless of innate sensitivity or resistance to anti‐PD‐1.

### 2.3 The CDK4/6i Priming Regimen Leads to Enhanced T Cell Infiltration, M1‐Like TAM Polarization, and Decreased Neutrophil Population in TME

**Figure 3:**
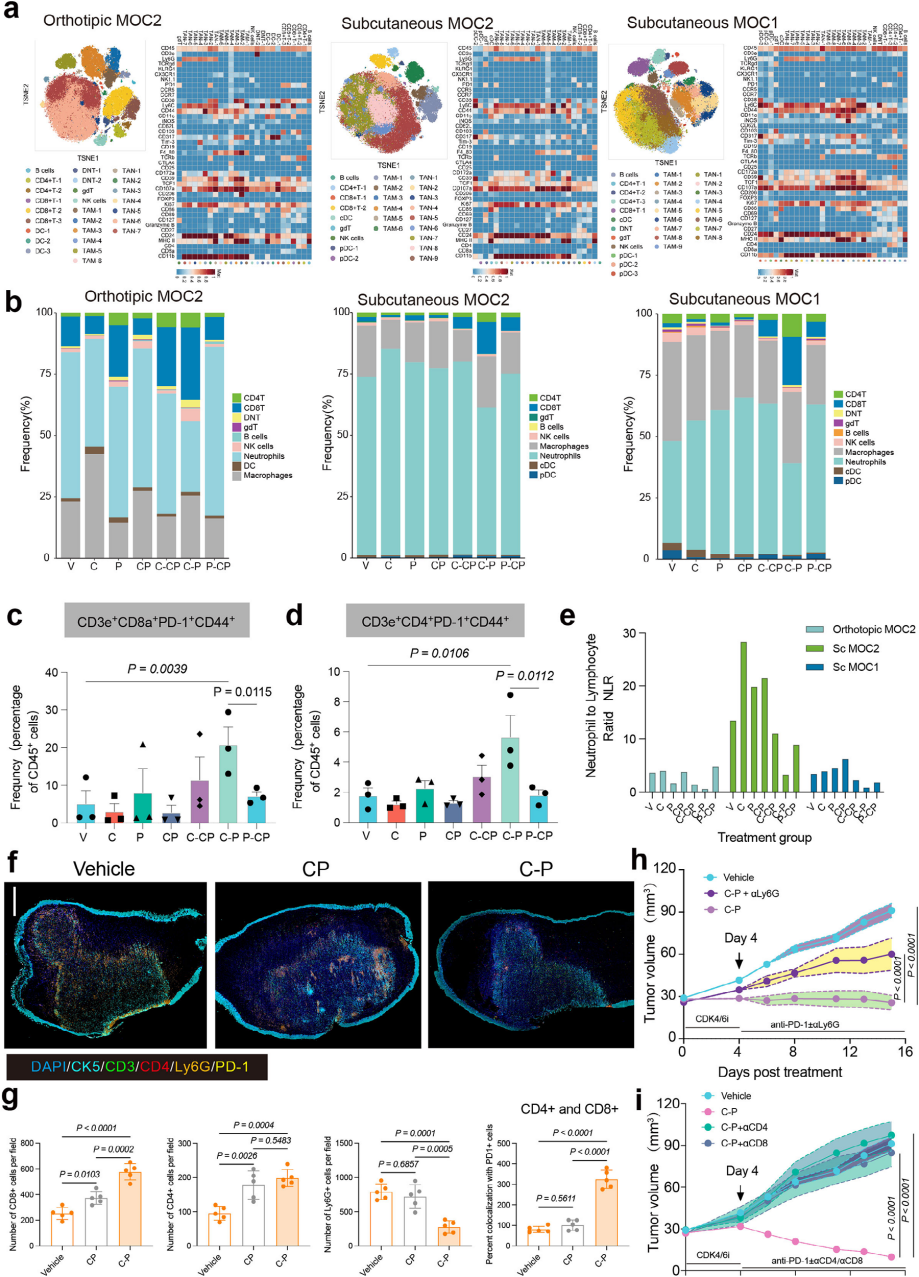
T cell expansion is predominantly associated with response to optimized therapy regimen. a) t‐SNE maps (left) of tumor‐infiltrating CD45^+^ cells analyzed by CyTOF in three indicated syngeneic subcutaneous or orthotopic tumor models at time points and in treatment regimens indicated in Figure 2g–j. Three pooled tumor tissues were collected from syngeneic subcutaneous or orthotopic tumor models at time points and in treatment regimens including: 1) vehicle, 2) CDK4/6i, 3) anti‐PD‐1, 4) CDK4/6i + anti‐PD‐1 (on d4 for orthotopic and d7 for subcutaneous tumor); 5) C‐PC, 6) C‐P, 7) P‐CP (on d11 for orthotopic and d13 for subcutaneous tumor). Heatmaps (right) showing the expression values of immune phenotypic protein markers normalized to the maximum mean value across subsets. b) Frequencies of immune cell types in the CD45^+^ population of three pooled tumor tissues collected from syngeneic tumor models at time points and treatment regimens indicated in Figure 2g–j. c,d) Frequencies of PD1^+^CD44^+^CD4^+^ and PD1^+^CD44^+^CD8^+^ in the CD45^+^ population of three syngeneic tumor models at time points and treatment regimens indicated in Figure 2g–j, n = 3, one‐way ANOVA test. e) NLR calculated as absolute neutrophil counts divided by absolute lymphocyte counts in three syngeneic tumor models at time points and treatment regimens indicated in Figure 2i–l. f,g) Representative images of mIF staining of CK5, Ly‐6G, CD4, CD8, PD‐1 in V, CP and C‐P of MOC2 orthotopic tumors and related histological quantification are shown. Scale bar, 1000 µm, n = 5, one‐way ANOVA test. h,i), MOC2 tumor‐bearing mice were treated with αLy6G, αCD4, αCD8, or isotype control 2 days prior to treatment with C‐P, then every 3 days until end of C‐P or vehicle treatment. Tumor volume was measured every 2 to 4 days. Data in (c, d, h, i) are presented as mean ± S.E.M. Data in g) are presented as mean ± S.D.

To identify changes in immune cell populations associated with the CDK4/6i priming before anti‐PD‐1 regimen, we sampled tumors from all seven treatment groups at the indicated time in mice harboring subcutaneous MOC1/2 or orthotopic MOC2 tumors (Figure 2b,e,f). Dissociated cells from each tumor models were then analyzed using immune cytometry by time‐of‐flight (CyTOF) assays (**Figure** **3**a). Among CD45^+^ cells, no difference in the proportions of CD4^+^ and CD8^+^ T cell populations were observed between the C, P, or CP treatments compared with vehicle controls (Figure 3b). However, these populations were not associated with efficacy in the treatment endpoint, as these proportions served as the baseline references for the corresponding priming regimens. By contrast, mice treated with the C‐PC, and to a greater extent the C‐P, CDK4/6i priming regimens displayed higher CD4^+^ and CD8^+^ T cell abundance versus either monotherapy and other treatment groups in all three tumor models (Figure 3b). In addition, increased percentages of PD1^+^CD44^+^ CD4^+^ or PD1^+^CD44^+^ CD8^+^ effector memory T cells were consistently observed in all three models (Figure 3c,d). Subsequent sub‐clustering of CD4^+^ T cells revealed that Tregs (CD25^+^) decreased while effector memory T cells (Tems) increased in the two CDK4/6i priming regimens (Figure S3a,b, Supporting Information). Sub‐clustering analysis CD8^+^ T cell populations showed that CDK4/6i priming led to reduced PD1^+^CD44^+^ CD8^+^ Tems levels (Figure S3c,d, Supporting Information), but this decrease was reversed following initiation of anti‐PD1 treatment in both orthotopic and subcutaneous MOC2 mice. Of note, terminal exhausted CD8^+^ T cells accumulated over time in the CDK4/6i and anti‐PD‐1 priming groups but not in the C‐P regimen (Figure S3c,d, Supporting Information), which led to the most extensive and durable tumor regression in the orthotopic and subcutaneous MOC2 models.

Among CD45^+^ cells in all treatment groups, tumor‐associated neutrophils (TANs) and tumor‐associated macrophages (TAMs) were the two most abundant clusters in all three tumor models. Interestingly, examination of the intratumoral neutrophil‐to‐lymphocyte ratio (NLR), a prognostic indicator of immunotherapy,^[^ ^24^ ^]^ revealed that both CDK4/6i priming groups had lower NLR than vehicle controls in the two MOC2 models, but increased in the concurrent (i.e., non‐priming) treatment groups in all three models (Figure 3e). Notably, the C‐P regimen led to greatest therapy‐associated decrease in NLR versus vehicle in all three models,(Figure 3e) which was further confirmed by immunofluorescence staining (Figure 3f,g; Figure S3e, Supporting Information). Cell typing by CyTOF also identified between six and nine TAM clusters in each of the three tumor models (Figure 3a), and reclustering of these TAMs uncovered M1‐like TAMs in 47.1% (8/17) and 26.7% (4/15) of TAM clusters in subcutaneous MOC2 and MOC1, respectively, but only in 18.8% (3/16) TAM clusters in orthotopic MOC2 (Figure S3f, Supporting Information). Subcutaneous MOC1 and MOC2, but not orthotopic MOC2, mice treated with C‐P exhibited the highest induction of M1‐like TAMs (Figure S3g, Supporting Information). Although neutrophils showed a pronounced reduction following C‐P treatment in all models, but still comprised a large proportion of intratumoral immune cells (Figure 3b). Given the above results, we hypothesized that depleting whole neutrophils might further augment the antitumor effects of C‐P treatment. We found that, in orthotopic MOC2 mice treated with anti‐Ly6G, C‐P treatment led to significantly lower antitumor efficacy compared to C‐P treatment in this model without neutrophil depletion (Figure 3h), suggesting that a subset of neutrophils could potentially promote or enhance the antitumor effects of this priming regimen. Similarly, neutralizing CD8^+^ or CD4^+^ T cells in orthotopic MOC2 mice abolished the therapeutic benefits of C‐P, i.e., the durability of tumor regression (Figure 3i; Figure S3h,i, Supporting Information). These results demonstrated that CDK4/6i priming followed by anti‐PD‐1 regimen eliminated tumor cells in a CD8^+^ and CD4^+^ T cell‐dependent manner.

### 2.4 CDK4/6i Priming Augments Progenitor‐Like Subpopulations in CD8^+^ Tex

**Figure 4:**
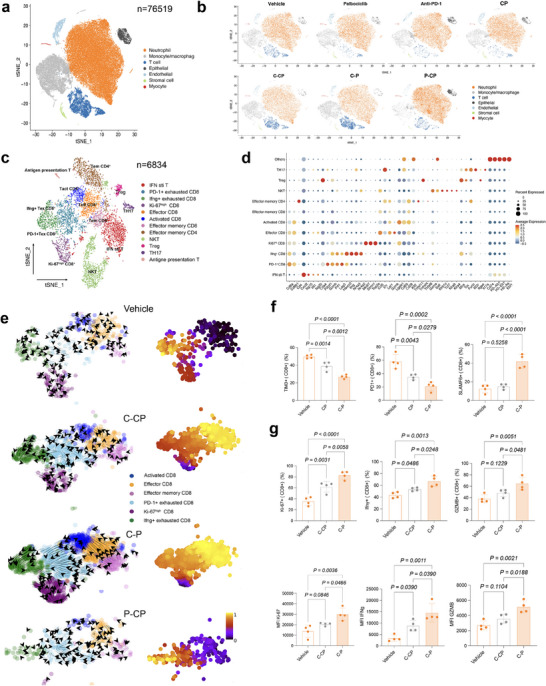
CD8^+^ Tex tended to form progenitor‐like Tex and retain proliferative and cytotoxic capacity under C‐P regimens. a) Maintype TSNE dimensionality reduction visualization of scRNA data in MOC2 syngeneic orthotopic tumor models at time points and in treatment regimens indicated in Figure S4a (Supporting Information). Three pooled tongue tumor tissues were collected from MOC2 syngeneic orthotopic tumor models at time points and in treatment regimens including: 1) vehicle, 2) CDK4/6i, 3) anti‐PD‐1, 4) CDK4/6i + anti‐PD‐1 (on d4); 5) C‐PC, 6) C‐P, 7) P‐CP (on d10). b) Maintype TSNE dimensionality reduction visualization by sample grouping. c) Visualization of TSNE dimensionality reduction for T cell subgroups. d) Visualization of T cell subgroups marker gene expression using a dot plot. e) Visualization of pseudotime analysis for CD8^+^ T cell subgroups using scVelo, including RNA velocity_embedding_stream and latent time. f) Quantification of flow cytometry of PD‐1, TIM3, SLAMF6 in CD8^+^ T cells from vehicle, CP, and C‐P groups (= 4 mice per group). g) Quantification of flow cytometry of Ki‐67, IFNγ, Granzyme B in CD8^+^ T cells from vehicle, C‐PC, and C‐P groups (n = 4 mice per group). Data are presented as mean ± S.D in (f) and (g). Statistical significance was determined by one‐way ANOVA test in (f) and (g).

To verify the above findings, we pooled cells from orthotopic MOC2 tumors in each time point in each treatment group for scRNA‐seq (Figure S4a, Supporting Information). Analysis of known lineage markers in high quality sequencing data from 76519 cells identified seven major cell types, including monocyte/macrophages (Mo/MF), T cells, TANs, epithelial cells, stromal cells, endothelial cells, and myocytes (**Figure** **4**a; Figure S4b and Table S2, Supporting Information). Consistent with CyTOF analysis (Figure 3a,b), TANs constituted the largest CD45^+^ cluster, followed by TAMs and T cells (Figure 4a).

We then quantified the percentages of the main cell types in all seven groups. In line with our above results, T cells and neutrophils showed the greatest change in proportions among all cell types following CDK4/6i priming regimens (Figure 4b; Figure S4c, Supporting Information); and the minor decrease in T cell populations associated with lead‐in doses of CDK4/6i (Figure S4c, Supporting Information), which could account for the lower T cell proliferation in these groups, was abolished by switching to or combining anti‐PD‐1. By d10, T cell levels were clearly elevated in C‐P‐ or C‐PC‐treated tumors relative to the CP and vehicle groups (Figure 4b; Figure S4c, Supporting Information). Graph‐based sub‐clustering of T cells by phenotype (n = 6834 cells) identified twelve T cell subpopulations, including six CD8^+^ clusters (one effector T, Teff; one activated T, Tact; one effector memory T, Tem; two exhausted T; Tex and Ki‐67hi T); two CD4^+^ clusters (Tem and Treg); antigen presenting T cells expressing Cd74, Cd83, etc.; Th17 cells; NKTs; and interferon‐stimulated T cells (IFN‐T) (Figure 4c,d; Figure S4d and Table S3, Supporting Information).

To better understand differences in T cluster developmental trajectory arising from each regimen, we conducted RNA velocity analysis of all CD8^+^ cells and found that two Tex subcluster located in the end portion of the trajectory in the vehicle and PD‐1 lead‐in groups, indicating a terminal exhausted state of CD8^+^. However, a strikingly opposite trajectory in CDK4/6i priming regimens wherein cells from two CD8^+^ Tex clusters and Ki67hi cells that comprised large proportions in the early and middle portions of the trajectory developed into other T cell clusters (Figure 4e; Figure S4e, Supporting Information). Recent work has uncovered distinct subsets of CD8^+^ Tex populations with functionally heterogeneous status, including terminal CD8^+^ Tex (Ttex) and progenitor CD8^+^ Tex (Tpex), that latter of which preferentially respond to ICB.^[^ ^6^, ^25^ ^]^ Based on the results of RNA velocity analysis, we hypothesized that CD8^+^ Tex cells were more likely to have a more progenitor exhausted phenotype following priming with CDK4/6i, consequently enhancing treatment response. Flow cytometry of T cell identity markers testing this possibility showed that expression of the memory/progenitor‐related gene, SLAMF6, was markedly elevated, while membrane expression of Ttex‐associated inhibitory receptors, PD‐1 and TIM3, decreased in the CDK4/6i priming groups compared with vehicle controls or other combination treatments (Figure 4f; Figure S4f, Supporting Information). Together, these data suggested that priming with CDK4/6i results in enrichment with the progenitor‐like CD8^+^ Tex subset.

### 2.5 Priming, but not Later Treatment, with CDK4/6i Augments T Cell Proliferation, Memory, and Effector Functions

Although both CDK4/6i priming regimens led to remarkable increases in T cell infiltration, we observed significantly slower tumor growth, and even regression, in C‐P‐treated tumor‐bearing mice compared with the C‐PC treatment group. Considering the well‐known effects of CDK4/6i in blocking cell cycle, we speculated that continuing exposure to CDK4/6i after priming could limit T cell proliferation and effector differentiation. GO analysis of upregulated genes in T cells from the C‐P and C‐PC groups revealed enrichment for pathways related to T cell differentiation, T cell proliferation, and T cell receptor signaling in the C‐P group (Figure S4g and Table S4, Supporting Information). As expected, CD8^+^ Tact and CD8^+^ Teff clusters displayed significantly higher enrichment scores for activated‐ and effector‐related pathways in the C‐P group (Figure S4h and Tables S5–S8, Supporting Information). Moreover, CD8^+^ Tex cells in C‐P group had higher expression of progenitor Tex signatures but lower terminally exhausted Tex signatures than the C‐PC group (Figure S4h and Tables S9 and S10, Supporting Information). It was especially noteworthy that all CD8^+^T clusters in the C‐P group had significantly higher T cell‐mediated anti‐tumor response scores, with the highest score in the Tex cluster, than corresponding clusters in the C‐PC group (Figure S4i and Table S11, Supporting Information). Consistent with these findings, flow cytometry analysis revealed that markers of proliferation (Ki67) and effector cytokine production (GZMB, IFNg) were significantly higher in tumor‐infiltrating CD8^+^T cells of the C‐P group relative to the C‐PC group (Figure 4g; Figure S4j, Supporting Information). These data demonstrated that applying CDK4/6i strictly as a priming agent could stimulate differentiation into proliferative and effector CD8^+^T cells in vivo, leading to the anti‐tumor effects observed in the C‐P regimen, while prolonged or later application, as in the C‐PC regimen, could attenuate this effect.

### 2.6 Stat5a Mediates the Antitumor Phenotype of CD8^+^ Tpex Cells

**Figure 5:**
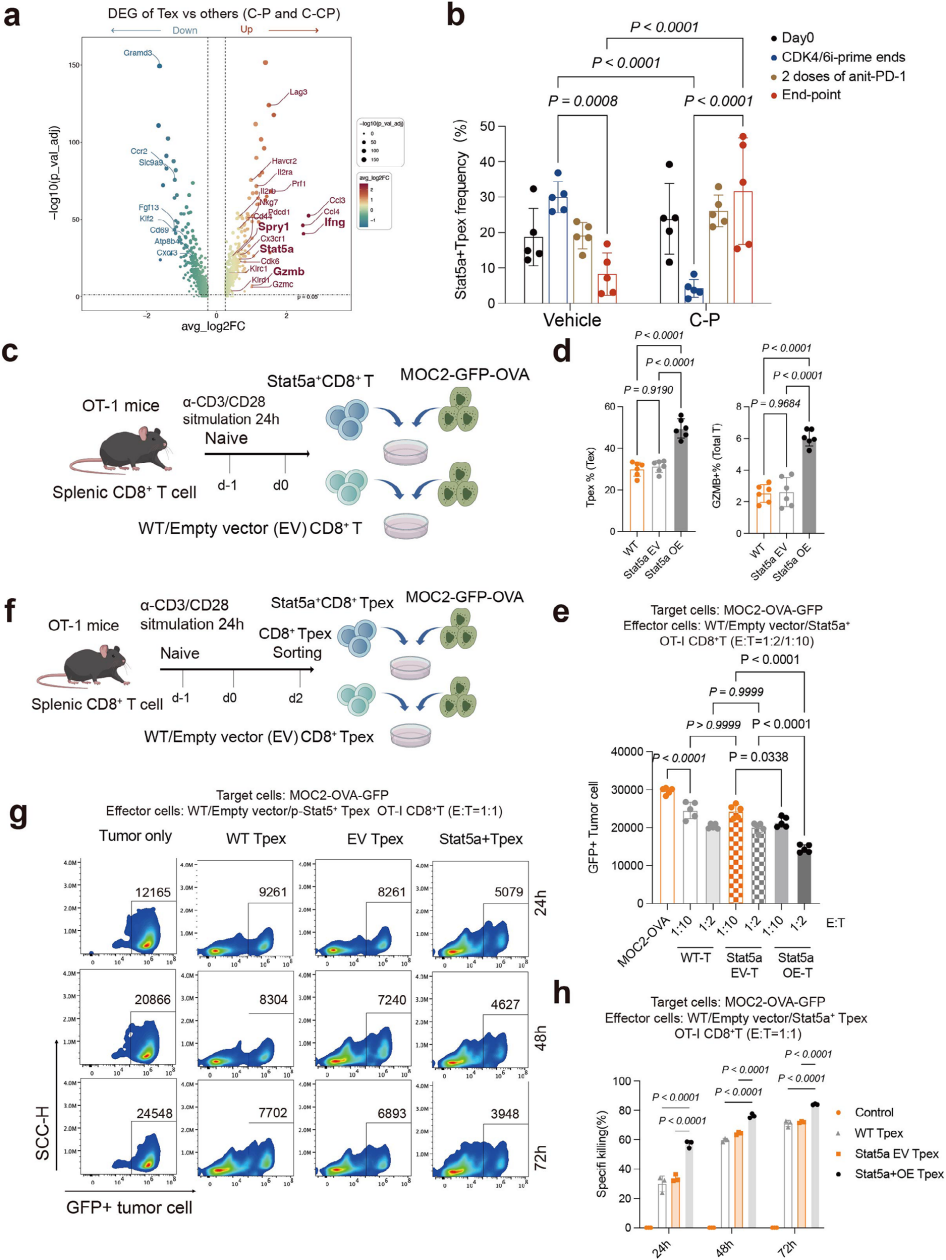
Stat5a mediates the functionally antitumor phenotype of CD8^+^Tpex cells. a) Volcano plot showing the DEG between CD8^+^ Tex and other CD8^+^ T cells in C‐P and C‐PC group. b) Bar charts showing the proportion of Stat5a^+^Tpex in the C‐P group and the vehicle group at various time points (n = 5). c) Experimental design. d) Quantification of flow cytometry analysis of frequencies of CD8^+^ Tpex, GZMB CD8^+^ cells in WT, Stat5a EV and OE groups (n = 6), two‐way ANOVA test. e) Specific cell killing of GFP+ target cells during the co‐incubation of MOC2‐OVA‐GFP and WT, Stat5a EV or Stat5a OE OT‐I CD8^+^ T‐cells for 48h under indicated E:T ratio (n = 5). two‐way ANOVA test. f) Experimental design. g,h) Absolute number of GFP^+^ target cells during the co‐incubation of MOC2‐OVA‐GFP and WT OT‐I‐Tpex, Stat5‐EV OT‐I‐CD8^+^Tpex or Stat5‐OE OT‐I‐CD8^+^Tpex at the indicated times (n = 3). The representative FACS plots for GFP^+^ target cells are shown (g). Quantification of specific killing percentage in indicated time is shown (h), (n = 3), two‐way ANOVA test. Data are presented as mean ±s.d in (b, d, e, h).

To investigate Tex cell‐intrinsic changes in gene expression that might drive the antitumor functional phenotype, we further identified DEGs between the CD8^+^Tex and other CD8^+^ subclusters under the two CDK4/6i priming regimens. This analysis yielded 858 total DEGs, including 440 upregulated genes (including *Klrd1*, *Klrc1*, *Gzmb*, *Stat5a*, *Spry1*, *Ifng*) and 418 downregulated genes (including *Ccr2*, *Fgf13*, *Klf2*, and *Cxcr3;* **Figure** **5**a; Table S12, Supporting Information). GO analysis showed that immune pathways (such as T cell activation and Leukocyte mediated cytotoxicity) were enriched in upregulated DEGs of CD8^+^Tex subclusters (Figure S5a and Table S13, Supporting Information). These results suggested that the CD8^+^ Tex population exerted more potent immune functions than other clusters, including CD8^+^ Tact or CD8^+^ Teff, under CDK4/6i priming regimens. We noticed that downstream targets of the IL2/IL15 signaling pathway, such as *Il2ra* (CD25), *Il15ra* (CD215), *Stat5a* and *Stat5b*, especially for the *Stat5a*, were preferentially expressed in Tex subclusters (Figure S5b, Supporting Information), which was consistent with previous reports that Tex cells with high CD25 expression might underlie tumor response to anti–PD‐1 and IL‐2 combination therapy.^[^ ^26^ ^]^ As Stat5a can regulate Tex cell differentiation, antagonize terminal exhaustion, and foster improved effector activity,^[^ ^27^ ^]^ we first conducted a longitudinal analysis to investigate the dynamic change of Tpex population, especially for the Stat5a^+^ subtype, under C‐P treatment overtime. In untreated tumors, Stat5a+Tpex cells exhibited an initial increase followed by a decline to low levels, which was effectively reversed by C‐P treatment (Figure 5b). Unexpectedly, C‐P treatment did not increase p‐Stat5 expression in PD1^+^TIM3^−^ or PD1^+^TIM3^+^ Tex cells in vitro (Figure S5c, Supporting Information), suggesting indirect mediation of p‐Stat5 expression in Tpex.

To examine the role of Stat5a in reprogramming Tex cells, we first conducted lentivirus‐mediated overexpression of Stat5a in OT‐I CD8^+^ T cells and validated the overexpression efficiency by western blotting (Figure 5c; Figure S5d, Supporting Information). Phenotypic characterization and tumor‐killing capacity between Stat5a‐overexpressing T cells with control groups were performed. The results demonstrated that Stat5a‐overexpressing T cells exhibited a significantly higher proportion of precursor exhausted T cells (Tpex) along with elevated expression levels of granzyme B (GZMB) compared to control cells (Figure 5d). These findings were consistent with the enhanced cytotoxic capacity observed in co‐culture experiments with MOC2‐OVA‐GFP tumor cells, where Stat5a‐overexpressing T cells demonstrated superior tumor‐killing efficacy relative to controls (Figure 5e). To further explore whether Stat5a overexpression on tumor‐specific CD8^+^ Tpex cells could directly enhance specific cell killing, Slamf6^+^PD‐1^+^TIM‐3^‐^CD8^+^ Tpex were sorted and Stat5a‐overexpressing CD8^+^ Tpex cells and vector controls were generated, and then co‐cultured with MOC2‐OVA‐GFP cells (Figure 5f). We also found that the Stat5a Tpex group showed significantly greater cell killing capacity than the vector controls (Figure 5g,h; Figure S5e, Supporting Information). Ex vivo cytotoxicity assays using reprogrammed Tex cells from C‐P treated tumors showed a significantly enhanced cytotoxicity capacity than vehicle‐derived Tex (Figure S5f,g, Supporting Information). All these results supported the likelihood Stat5a mediates the enhanced response to immunotherapy in CD8^+^ Tpex cells, contributing the antitumor efficacy of C‐P regimen.

### 2.7 Sell^hi^ Neutrophils Induce Functional Stat5a^+^CD8^+^Tpex Cells via IL15‐Stat5a Axis

As the C‐P regimen could not directly induce p‐Stat5^+^CD8^+^Tpex in vitro (Figure S5c, Supporting Information), we next investigated whether other TME component participated in this C‐P‐responsive Tpex phenotype transition in vivo. As Il15ra is preferentially expressed in Tex (Figure S5b, Supporting Information), and IL15 cytokine binding with Il15ra could generate and maintain memory T cells,^[^ ^28^ ^]^ while activating the canonical downstream STAT family,^[^ ^29^ ^]^ including STAT5. We hypothesis that cells producing IL15 could effectively induce p‐Stat5^+^CD8^+^Tpex in vivo. Although IL15 was reported to be secreted mainly by stromal and myeloid cells,^[^ ^30^ ^]^ especially for DCs, our ScRNA‐seq data analysis and mIF (Figure S6a,b, Supporting Information) indicated that IL15 expression was found predominantly distributed in neutrophil clusters. Of note, although total neutrophils decreased following CDK4/6 priming (Figure 4b), and neutrophil depletion with anti‐Ly6G could not enhance response to C‐P (Figure 3g), we speculated IL15 producing neutrophils might contribute to the antitumor activity of CDK4/6i priming.

**Figure 6:**
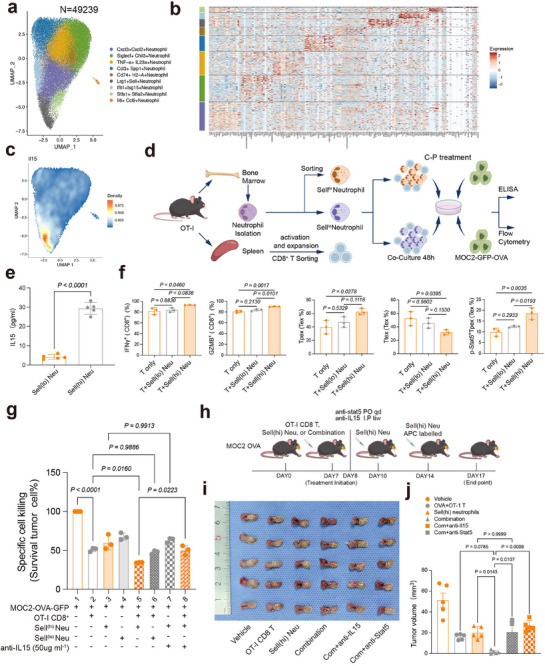
Sell^(hi)^ neutrophils induced CD8^+^Tpex cells through IL15‐Stat5a axis. a) UMAP dimensionality reduction visualization of neutrophil clusters. b) Visualization of marker gene expression for neutrophil subgroups using a pheatmap. c) UMAP visualization of gene expression density for ll15 in neutrophil subgroups. d) Experimental design. e) IL15 secretion measured by ELISA in Sell^(hi)^ and Sell^(lo)^ neutrophil supernatant. One‐way ANOVA test. f) Bar plots showing the frequencies of Tpex, Ttex, p‐Stat5^+^ Tpex, IFNγ^+^, and GZMB^+^OT‐I CD8^+^ T cell in indicated treatment groups, one‐way ANOVA test. g) Specific cell killing of GFP^+^ target cells during the co‐incubation of MOC2‐OVA‐GFP, sorted Sell^(hi)^ and Sell^(lo)^ neutrophils and CD8^+^ T‐cells from OT‐I mice under indicated treatments and at the indicated times (n=3), one‐way ANOVA test. h) Experimental design of in vivo adoptive cell transfer using OT‐I CD8^+^ cells and neutrophils. i,j), Tumor images and volumes of in vivo experiment using MOC2‐OVA tumor orthotopic model treated with indicated treatments. Sell^(hi)^ and Sell^(lo)^ neutrophil population were defined as CD45+/CD11b+/Ly6C‐Ly6G+Sell+ and CD45+/CD11b+/Ly6C‐Ly6G+Sell‐. Data in (e‐g, j) are presented as mean ± S.D.

To identify the subset of IL15‐producing neutrophils, graph‐based Sub‐clustering identified nine neutrophil subpopulations (n = 49239 cells) (**Figure** **6**a,b), including Cxcl3^+^, Siglecf^+^, Tnf‐α^+^, Ccl3^+^, Cd74^+^, Sell^+^, Ifit1^+^, Stfa1^+^, and Il6^+^ subclusters. As IL15 were dominantly expression in Sell^+^ neutrophils (Figure 6c), reportedly accumulate in tumors during immunotherapy response and are associated with better treatment outcomes, we selected Sell^(hi)^ neutrophils as a proxy for IL15 producing neutrophils in subsequent experiments. Sell^(hi)^ and Sell^(lo)^ neutrophils were isolated and ELISA detection in culture medium indicated that IL15 secretion was significantly higher in Sell^(hi)^ neutrophils compared to Sell^(lo)^ compartment (Figure 6d,e). We then investigated whether IL15‐secreted Sell^(hi)^ neutrophils could directly induce the pStat5 Tpex and affected tumor killing T‐cell activity in co‐cultures of OVA‐tumor‐activated OT‐I CD8 T cells (1:1 ratio; 48 h) under C‐P treatments. The Tpex subset showed elevated p‐Stat5 expression and GZMB production compared to corresponding Sell(lo) control cultures, with a trend toward increased IFNγ production (Figure 6f; Figure S6c, Supporting Information). Cytolytic activity assays indicated that combination of neutrophils, especially for the Sell(hi) subset with T cells demonstrated a significant increased killing capacity compared with T cells alone, but not the case for Sell(lo) subset (Figure 6g; Figure S6d, Supporting Information). We also found that all blockade groups using anti‐IL15 antibody or anti‐stat5 treatments largely attenuated the synergistic cytotoxic effects in the Sell(hi) neutrophil combined with OT‐I T cells while had no effects on Sell(hi) neutrophil cytotoxicity alone (Figure 6g; Figure S6d, Supporting Information).

Subsequently, we sought to test, in vivo, the synergistic effects of sell(lo) or sell(hi) neutrophils with T cells and their dependence on IL15‐Stat5a axis. We first administered Sell(hi) neutrophils, by adoptive transfer alone or with OT‐I CD8 T cells to mice bearing MOC2‐OVA tumors (Figure 6h). Consistent with the in vitro results, we found that Sell(hi) neutrophils combined with OT‐I CD8 T cells showed a significant stronger antitumor effect than either monotherapy alone and blocking IL15‐Stat5a axis significantly dampened this synergistic effect (Figure 6i,j). Of note, we identified fluorescently labeled Sell(hi) neutrophils in treated tumors (Figure S6e, Supporting Information), further indicating that Sell(hi) neutrophils successfully infiltrate and play a role in the responding tumor microenvironment. We also examined the levels of various immune cells and found that antibodies or inhibitor targeting IL15‐Stat5a axis did not elicit any significant changes of frequencies or functions in other immune cell types including NK cells, DCs or M1/M2 macrophages (Figure S6f, Supporting Information). To further investigate the potential contributions of other neutrophil subpopulations to the antitumor response, we also administered Sell(lo) neutrophils, by adoptive transfer alone or with OT‐I CD8 T cells to mice bearing MOC2‐OVA tumors and no significant synergistic tumor growth inhibition was observed in combination groups (Figure S6g–i, Supporting Information). Moreover, there are no differences in frequencies of Tpex or Stat5a^+^Tpex between OT‐I CD8 T cells and combination groups (Figure S6j, Supporting Information). Taken together, all these data reinforce the fact that Sell^(hi)^ neutrophils enhanced p‐Stat5^+^CD8^+^Tpex‐mediated tumor cell killing through IL15‐stat5a axis.

### 2.8 Blocking the IL15‐stat5a Axis Decreases Antitumor Efficacy of C‐P Regimen

**Figure 7:**
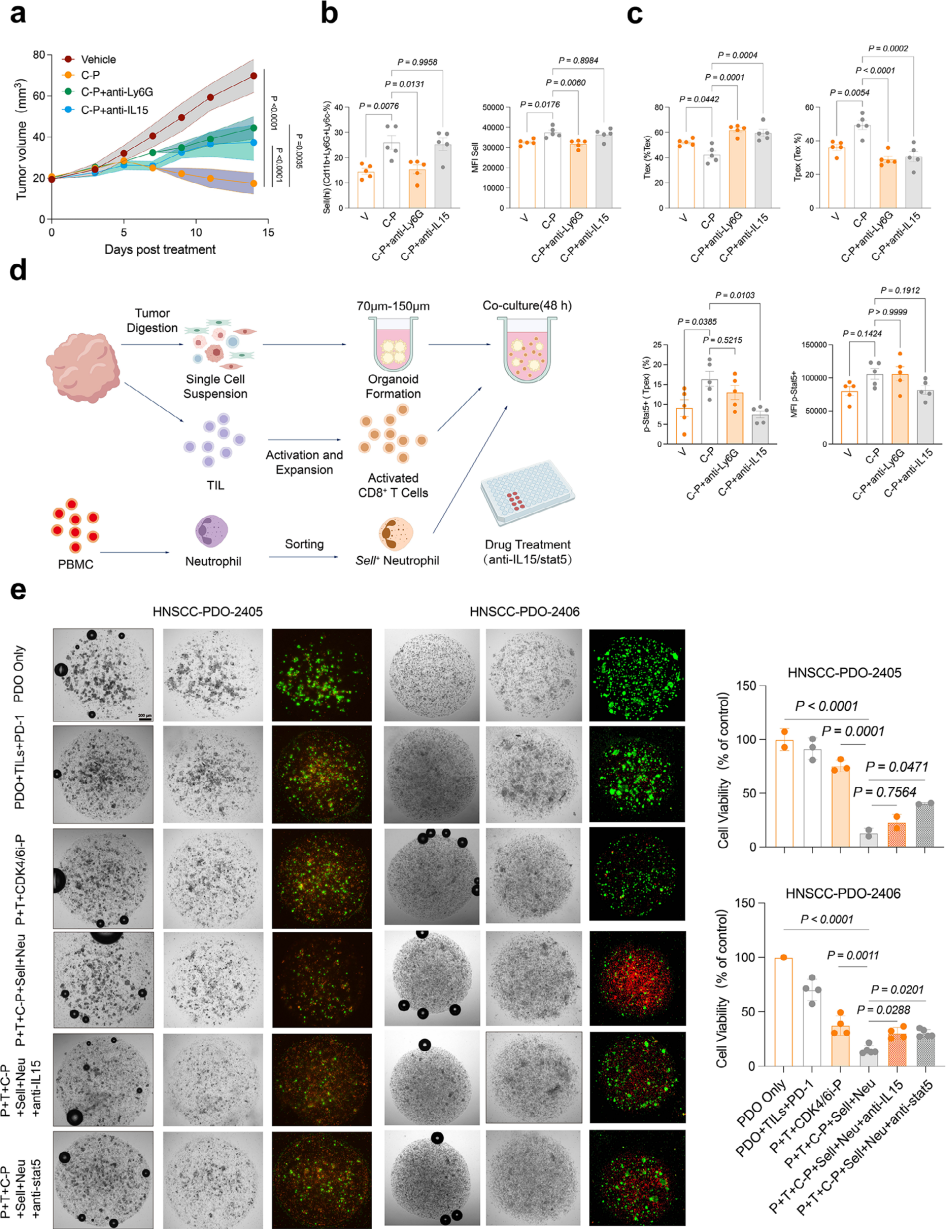
Blocking IL15‐Stat5a signaling dampens antitumor activity of C‐P regimen. a) Mean tumor growth curves showing tumor volume in mice treated with IgG, C‐P regimen, C‐P regimen plus anti‐IL15 antibody and C‐P regimen plus anti‐Ly6G antibody. (n = 5 mice per group), two‐way ANOVA test. b) Frequencies of Sell^(hi)^ neutrophils in indicated treatment groups, n=5, one‐way ANOVA test. c) Frequencies of Tpex, Ttex and p‐Stat5^+^ Tpex in indicated treatment groups, n=5, one‐way ANOVA test. d) Experimental design. e) Representative bright fields and Calcein/PI fluorescence staining images of Organoid/TIL coculture assays derived from two HNSCC patients (left). Relative cell viability of each treatment group (right). Data in Data in (e) are presented as mean ± s.d. P+T: PDO+TIL; C‐P: CDK4/6 lead‐in before PD‐1.

As blocking IL15‐stat5a axis significantly dampened the Sell^(hi)^ neutrophil‐mediated tumor cell killing capacity of p‐Stat5^+^ Tpex, we next assessed whether blocking this axis also reduced the antitumor efficacy of the C‐P regimen in MOC2 tumor‐bearing mice in vivo (Figure S7a, Supporting Information). Mice treated with C‐P plus anti‐IL15 or anti‐Ly6G antibody had less inhibition of tumor growth than those treated with C‐P alone (**Figure** **7**a; Figure S7b, Supporting Information). Consistent with this decreased survival, Ly6G blockade attenuated the increase in Sell^(hi)^ neutrophil proportions observed in the C‐P group (Figure 7b; Figure S7c, Supporting Information), whereas IL15 blockade had no such effect on Sell^(hi)^ neutrophil induction. At the same time, the Ttex proportion significantly decreased while the Tpex subset expanded in C‐P treatment groups compared with vehicle controls; both Ly6G‐ or IL15 blockade resulted in the opposite effect (Figure 7c; Figure S7c, Supporting Information). Within the Tpex subpopulation, C‐P treatment resulted in increased Stat5 expression, and this effect was abolished in the anti‐IL15 group (Figure 7c; Figure S7c, Supporting Information). Of note, enhanced proliferative or cytotoxic capacity of CD8+ T cells (i.e., upregulated Ki‐67, IFNγ, Granzyme B expression, etc.) of the C‐P group was dampened in the C‐P + anti‐IL15 and C‐P + anti‐ Ly6G groups (Figure S7d,e, Supporting Information).

We next examined the potential for clinical application of C‐P regimens in HNSCC through co‐culture of patient‐specific tumor‐reactive T cells (i.e, autologous tumor‐infiltrating lymphocytes, TILs) and Sell^(hi)^ neutrophils in Matrigel with HNSCC‐PDOs (Figure 7d). Two patients (patient 2405 and 2406) were included for C‐P response evaluation. Immunofluorescence staining revealed that autologous TILs showed minimal, non‐significant changes in organoid killing upon anti‐PD‐1 treatment in patient 2405‐derived organoid (Figure 7e), indicating an intrinsic immune‐resistant state in this patient. However, PI/Calcein staining showed that addition of Sell^hi^ neutrophil isolated from PBMC of donor patient to C‐P resulted in a striking decrease in tumor cell viability, leading to a synergistic, significant increase in apoptosis levels in 2405 organoids, whereas blockade of IL15 or the IL15 target, Stat5a, impaired this synergistic effect (Figure 7e). However, we observed anti‐PD‐1 treatment elicited a significant reduction of cell viability in another coculture system from donor patient 2406, which represented a relatively intrinsic sensitivity to immunotherapy (Figure 7e). As we observed in patient 2405, C‐P regimen combined Sell^hi^ neutrophil also showed the most extensive viability reduction of patient 2406 organoid and IL15 signaling blockade also significantly rescued this effect. These cumulative data highlighted the importance of the IL15‐stat5 axis in enhancing the antitumor efficacy of the C‐P regimen.

## 3 Discussion

Cyclin D1‐CDK4/6 signaling has been associated with immune infiltration and responses.^[^ ^3^, ^4^ ^]^ Targeting cyclin D1‐CDK4/6 signaling with CDK4/6i can affect antitumor responses by influencing both tumor cells and the TME.^[^ ^31^ ^]^ However, mechanisms underlying the synergistic effects of CDK4/6 inhibitors and ICB and optimal combination strategies remain unclear. We demonstrated that activation of the cyclin D1‐CDK4/6 pathway intrinsic to tumor cells results in reduced T‐cell infiltration, proliferation, and responses. Importantly, we found that priming with CDK4/6i before anti‐PD‐1 treatment enhances the synergistic antitumor effects through rejuvenation of the Tex state via an Sell^(hi)^ neutrophil‐mediated IL15‐STAT5 axis (Figure S8, Supporting Information).

The five‐year overall survival (OS) rate for recurrent/metastatic head and neck squamous cell carcinoma (R/M HNSCC) remains dismal, generally below 10%, with a median OS of 6–12 months and progression‐free survival (PFS) of 2–6 months.^[^ ^32^ ^]^ This poor prognosis is attributed to intrinsic chemoresistance, immunosuppressive microenvironments, and frequent relapse post‐standard therapies. Palbociclib plus cetuximab demonstrates modest survival benefits in platinum‐resistant, cetuximab‐naïve HPV‐negative patients, achieving a median OS of 9.5 months and 1‐year OS rate of 36.5% (vs 6.0 months with cetuximab monotherapy).^[^ ^33^ ^]^ However, its efficacy diminishes in cetuximab‐refractory cohorts (median OS: 6.3 months). While CDK4/6 inhibitor combinations with PD‐1 monoclonal antibodies have only recently gained attention in head and neck squamous cell carcinoma (HNSCC) with no mature 5‐year overall survival (OS) data available, such strategies have been extensively explored in other malignancies including melanoma and breast cancer. Although combination therapies are typically administered simultaneously, the “priming strategy” using CDK4/6 inhibitors—short‐term pretreatment to synchronize tumor cell cycles and enhance sensitivity to subsequent therapies—has emerged as a focus in HNSCC clinical research. Studies indicate that CDK4/6 inhibitors induce G1‐phase arrest in HNSCC cells, followed by synchronized S‐phase entry upon withdrawal, thereby amplifying the cytotoxic effects of chemotherapy (e.g., cisplatin, 5‐FU) or radiotherapy.^[^ ^34^ ^]^ Previous studies have investigated administering targeted inhibitors or chemoradiotherapy in sequence with ICIs.^[^ ^22^, ^35^ ^]^ Only a 23.1% objective response rate was observed in a clinical trial evaluating the safety and efficacy of concurrent ribociclib (CDK4/6i) and spartalizumab (anti‐PD‐1) for HNSCC. From a clinical perspective, staggering these regimens in a sequential manner may reduce high‐grade toxicities while preserving the immune‐potentiating effects of CDK4/6 inhibitors. These findings suggest that the efficacy of distinct sequential modalities may be due to tumor‐specific characteristics and drug‐specific effects of the TME.

Currently, PD‐1 antibody‐based combination therapy has been approved as a first‐line standard of care only for recurrent or metastatic (R/M) HNSCC, one limitation is that in vivo tumor models in this study were all established as orthotopic or subcutaneous tumors, and how these models represent R/M HNSCC remains unclear. Further validation using humanized patient‐derived xenografts (PDXs) models established from R/M HNSCC patients might provide more clinically relevant evidence of C‐P efficacy in the setting of R/M HNSCC. Most strategies involving CDK4/6i combined with ICB in preclinical and clinical investigations have focused on concurrent administration. This approach may limit the maximal antitumor response. Compared with the approved combination regimen like the use of CTLA‐4 and PD‐1 inhibitors in melanoma,^[^ ^36^ ^]^ which showed limited efficacy in other cancer types, sequential therapy using CDK4/6i and PD‐1 inhibitors may reduce the side effects associated with the concurrent use of two immune checkpoint inhibitors, such as immune‐related adverse events (irAEs) and have shown efficacy in various cancers, such as breast cancer,^[^ ^37^ ^]^ and sequential PD‐1 therapy may expand their application scope. In our studies, we found that early priming with CDK4/6i significantly enhances the efficacy of subsequent anti‐PD‐1 therapy compared to concurrent administration or anti‐PD‐1 therapy. These results suggest that a sequential modality of CDK4/6i followed by anti‐PD‐1 therapy should be further confirmed in a clinical setting, especially for the neoadjuvant therapy.

Recent studies have demonstrated the functional heterogeneity of CD8^+^ Tex.^[^ ^25^, ^38^ ^]^ Research indicates that Tpex positively correlates successful immunotherapy,^[^ ^6^, ^39^ ^]^ and Tpex were enriched in regional lymph nodes compared to tumors.^[^ ^40^ ^]^ However, this ICB responsiveness of Tpex was disrupted in metastatic lymph nodes, suggesting that a protective niche away from the TME is needed to preserve Tpex for sustaining an antitumor response. Enhancing the pool size of Tpex or overcoming the unfriendly niche for Tpex preservation through optimized combination therapy remains elusive. CDK4/6i have been proposed to establish a tumor‐specific memory CD8^+^ T‐cell pool,^[^ ^12^, ^13^ ^]^ thereby allowing for subsequent administration of immune checkpoint blockade. However, the joint impacts on T cell evolution have not been investigated. Our study revealed the progenitor‐like CD8^+^ Tex cells within the TME of CDK4/6i priming groups, which were associated with effective antitumor response.^[^ ^41^ ^]^ Further investigations are warranted to determine the impact of CDK4/6i priming regimens on lymphatic metastasis prevention and Tpex dynamics between draining lymph nodes (dLN) and primary tumors.

Currently, mechanisms underlying the programming of Tpex are not well understood. Hence, we showed that Stat5a was significantly upregulated in CD8^+^ Tpex cells, primarily within the TME of CDK4/6i priming groups. Previous studies have shown that Stat5a opposes the transcription factor Tox, redirecting exhausted CD8^+^ T cells toward durable effector‐like/exhaustion‐resistant states, enhancing polyfunctionality and antitumor immunity.^[^ ^27^ ^]^ Neutrophils are generally pro‐tumorigenic; however, recent research revealed that both anti‐ and pro‐tumor neutrophils coexist within tumors without differentiating.^[^ ^42^, ^43^ ^]^ Here, we identified an Sell^(hi)^ neutrophil cluster that increased predominantly in CDK4/6i monotherapy or priming groups. These neutrophils preferentially secreted IL15, contributing to the overexpression of Stat5a in Tpex under CDK4/6i priming regimens. We confirmed the functionality of these Sell^(hi)^ neutrophils in mediating specific antitumor efficacy using a neutrophil‐T‐cell‐tumor co‐culture system. Our work demonstrates the impact of CDK4/6i or CDK4/6i priming regimens on the induction of Tpex and their connection with anti‐tumor neutrophils. A systematic analysis of neutrophils in collected PBMCs^[^ ^44^ ^]^ might better explain the source of intra‐tumoral Sell^(hi)^ neutrophils under CDK4/6i priming regimens.

Furthermore, additional limitations exist in this study. First, our preclinical models were based on cancer cell lines, we did not include genetically engineered mouse models or humanized patient‐derived xenografts. However, for our preclinical approach, the use of organoid‐T cell co‐culture model might aid further investigation of CDK4/6i priming before anti‐PD‐1 clinically. Second, we did not focus on the impact of CDK4/6i priming regimens on preventing lymphatic metastasis control, which frequently occurred in HNSCC patients.^[^ ^41^ ^]^ Application of CDK4/6i priming regimens on an 4NQO‐induced carcinogenesis model in immunocompetent mice and analysis dynamic of draining lymph node (dLN) warrant further studies. In addition, the patient cohort analyzed here was relatively small, which limits the generalizability of the findings and calls for validation in larger, stage‐stratified cohorts. Third, owing to the higher density of neutrophils in peripheral blood,^[^ ^44^ ^]^ a systematic analysis of neutrophil in collect PBMCs might better explained the resource of intratumoral Sell^(hi)^ neutrophil underlying CDK4/6i priming regimens.

Overall, while there are several limitations to our study which deserve further exploration and clinical validation, we nevertheless provide strong evidence that CDK4/6i priming before anti‐PD‐1 therapy is a promising sequential modality in HNSCC and IL15‐secreted Sell^(hi)^ neutrophils induce Stat5^+^ progenitor CD8^+^ T cells is functionally involved in the generation of response. Future studies employing clinically relevant R/M HNSCC models, such as humanized PDXs, will also be important to further establish the translational potential of this sequential regimen.

## 4 Experimental Section

### Ethics Statement

The ethical committee of Ninth People's Hospital, Shanghai Jiao Tong University School of Medicine (SH9H‐2021‐T352‐2) approved the collection and use of human HNSCC samples. All mouse experiments complied with National Institutes of Health guidelines and were conducted with approval from the Institutional Animal Care and Use Committee (IACUC) of Ninth People's Hospital, Shanghai Jiao Tong University School of Medicine (SH9H‐2023‐A866‐1).

### Patient Samples

Tumor tissues were obtained from patients at Ninth People's Hospital, Shanghai Jiao Tong University School of Medicine, with written informed consent from each participant. The hospital's pathology department confirmed all surgically resected tumor samples and biopsies as malignant HNSCCs. Immediately after surgery, tumor specimens were collected in Dulbecco's modified Eagle's medium (DMEM, Gibco) supplemented with 1% penicillin‐streptomycin (PS, Gibco) and transported them on ice to the laboratory. To eliminate black secretions, the tissues were washed at least twice with DMEM containing 1% PS and a 1× gentamicin‐amphotericin solution (Gibco). After carefully removing excess muscle and bone, the tissues were cut into small fragments for organoid generation and TIL isolation.

To dissociate the tumor tissue, mild enzymatic digestion was performed using a Human Tumor Dissociation Kit (Miltenyi). First, the fragments were processed for 1 min with the MACS Dissociator (Miltenyi), then transferred them to an orbital shaker at 37 °C for 20–30 min. Tumor cells were collected by centrifugation at 1500 rpm for 5 min. Finally, ≈1 × 10⁴ cells were mixed with Matrigel (NEST Biotechnology) and seeded them into prewarmed, flat‐bottom 24‐well cell culture plates (NEST Biotechnology).

### Animals and Animal Experiments

Male C57BL/6 mice used in this study were obtained from Cyagen Bioscience Inc., while CD45.1 OT‐I mice, sourced from the Jackson Laboratory, were used for OT‐I CD8⁺ T cell isolation. All procedures adhered to National Institutes of Health guidelines. Mice were euthanized if they displayed signs of illness, lost more than 20% of their initial body weight, or developed subcutaneous tumors that were ulcerated or exceeded 2000 mm^3^, ensuring that tumor burden remained within ethical limits. For syngeneic subcutaneous tumor models, C57BL/6 mice were injected with 4 million MOC1 cells or 1 million MOC2 cells into the flanks. Once tumors reached 100–150 mm^3^, the mice were randomly assigned to experimental groups. In orthotopic tumor models, C57BL/6 mice were injected with 0.5 million MTCQ1 cells, 0.5 million MOC1 cells, or 0.05 million MOC2 cells into the tongue. Mice were randomly allocated to experimental groups on day 3 for MOC2 and day 5 for MTCQ1 and MOC1. Tumors were measured every three days using calipers and calculated tumor volumes using the formula (length × width^2^)/2.

Sample sizes for animal experiments were determined based on variability observed in preliminary studies, which were reviewed and approved as part of the IACUC protocol. The IACUC protocol also defined the criteria for stopping data collection. If a mouse died from causes unrelated to tumor burden, it was excluded from the analysis. Mice received the CDK4/6 inhibitor palbociclib (100 mg kg^−1^ day^−1^, Selleck) via oral gavage. For the CDK4/6i priming strategy, CDK4/6i (100 mg kg^−1^, orally, daily) was administered from day 0 (randomization) to day 4 for orthotopic tumors and from day 0 to day 7 for subcutaneous tumors after inoculation. In orthotopic models, CDK4/6i priming began on days 3–4 post‐inoculation, whereas in subcutaneous models, treatment started once tumors reached 100–150 mm^3^. Mice received intraperitoneal injections of anti‐PD‐1 (200 µg per mouse, BioXcell), anti‐IL15 (200 µg per mouse, BioXcell), anti‐Ly6G (200 µg per mouse, BioXcell) or an equivalent dose of isotype IgG control, along with cisplatin (5 mg kg^−1^, Selleck), twice per week. Additionally, STAT5‐IN‐1 (10 mg kg^−1^, Selleck) was administered orally on a daily basis. In Figure 3g, anti‐CD8 (200 µg per mouse, BioXcell), anti‐CD4 (200 µg per mouse, BioXcell), anti‐Ly6G (200 µg per mouse, BioXcell), or an equivalent dose of isotype IgG control was injected intraperitoneally on day ‐1, day 0, and every three days thereafter. On the specified days in each in vivo experiment (Figure 2a–d; Figure S4a, Supporting Information), tumors were excised, mechanically minced them, and enzymatically digested them into single‐cell suspensions using a tumor dissociation kit (Miltenyi) and the gentleMACS Dissociator (Miltenyi). Then the samples were prepared for CyTOF staining and sorted live CD45⁺ cells for single‐cell RNA sequencing when applicable.

### Cell Lines and Primary Tumor Cell Cultures

MOC1 (RRID:CVCL_ZD32) and MOC2 cell line (RRID:CVCL_ZD32), obtained from Kerafast (USA) were cultured in IMDM (Gibco) supplemented with 10% fetal bovine serum (FBS, NEST Biotechnology) and 1% penicillin‐streptomycin (PS, Gibco). MOC1 and MOC2 with distinct immunotherapy sensitivity were selected for comparison CDK4/6i priming modalities. Patient‐derived cell models, previously established in the study, were maintained in DMEM/F12 containing 10% FBS, 1% PS, insulin (5 µg mL^−1^, Sigma–Aldrich), amphotericin B (250 ng mL^−1^, Gibco), gentamicin (10 µg mL^−1^, Gibco), cholera toxin (0.1 nm, Sigma–Aldrich, Gibco), epidermal growth factor (EGF, 0.125 ng mL^−1^, PeproTech), hydrocortisone (25 ng mL^−1^, Sigma–Aldrich), and the ROCK inhibitor Y‐27632 (10 µm, Selleck). All cell cultures were incubated at 37 °C in a humidified atmosphere with 5% CO_2_. All cell lines were authenticated and confirmed they were free of mycoplasma contamination.

### Isolation, Viral Transduction, and Killing Assays for OT‐I CD8^+^ T Cells and OT‐I CD8^+^ Tpex Cells

To isolate OVA‐specific CD8⁺ T cells, spleens were harvested from OT‐I transgenic mice (6–8 weeks old) and gently dissociated them using a 70‐µm nylon cell strainer (BD Falcon). Then naïve OT‐I CD8⁺ T cells were isolated using CD8 (TIL) MicroBeads (Miltenyi) following the manufacturer's protocol. Purified CD8⁺ T cells (>90% purity) were seeded and activated in vitro for 24 h with 5 µg mL^−1^ CD3 monoclonal antibody (eBioscience) and 5 µg mL^−1^ Ultra‐LEAF purified anti‐mouse CD28 (BioLegend). Total splenocytes were cultured in 1 mL of T cell medium, consisting of RPMI (Gibco) supplemented with 10% FBS, 1% penicillin‐streptomycin, 1% nonessential amino acids (Gibco), 1% sodium pyruvate (Gibco), and 25 µm β‐mercaptoethanol (Gibco). Cells were incubated at 37 °C in a humidified 5% CO_2_ atmosphere and allowed to expand for 5–7 days. To isolate PD‐1⁺TIM‐3⁺SLAMF6⁺ TPEX cells, the OT‐I CD8⁺ T cells were collected and stained with antibodies against PD‐1, SLAMF6, and TIM‐3. Then the cells were sorted using a CytoFLEX (Beckman) flow cytometer. The coding sequence of mouse Stat5a (NM_011488.3) was subcloned into the pHBLV‐CMV‐MCS‐3flag‐EF1‐T2A‐Puro vector (Hanbio Tech) and co‐transfected this construct into 293T cells along with the packaging plasmid psPAX2 and the envelope plasmid pMD2.G to produce lentivirus. Viral transduction was performed by spin‐infection at 900 g at 25 °C for 3 h, using 10 µg mL^−1^ polybrene (Sigma–Aldrich). After transduction, CD8⁺ T cells were cultured as described above for 3 days. The purity of the transduced cells was assessed (Figure S9 and S5e, Supporting Information). MOC2‐OVA‐GFP target cells was incubated with either Stat5a⁺CD8⁺ T/Stat5a⁺CD8⁺ TPEX or EV CD8⁺ T/EV CD8⁺ TPEX at the indicated effector‐to‐target (E:T) ratios, in the presence of rIL‐2 (200 U mL^−1^). Initially, T cells and MOC2‐OVA‐GFP target cells were mixed (E:T = 1:1) in 48‐well flat‐bottom plates. GFP+ cell numbers were quantified by flow cytometry at the designated co‐incubation time points.

### Organoid‐TIL Co‐Culture and Drug Treatment—Generation of the HNSCC Organoid

HNSCC organoids was generated and cultured following established protocols with slight modifications. Briefly, fresh HNSCC tumor tissues were dissected into 0.5–1 mm piece and enzymatically digested using 1.5 mg mL^−1^ collagenase IV (Sigma–Aldrich), 10 mg mL^−1^ hyaluronidase type IV (Sigma–Aldrich), and 1 mg mL^−1^ DNase I (Sigma–Aldrich). The digested cells were then embedded in Matrigel. After allowing the Matrigel to solidify for 20 min at 37 °C, cells were overlaid with human HNSCC organoid medium, with medium changes occurring every three days. During the first two weeks of culture, the medium with 1x Primocin (Invivogen) was supplemented to prevent microbial contamination. Organoids were passaged weekly by treating them with TrypLE Express (GIBCO) for 5–10 min at 37 °C to dissociate the organoids into single cells, followed by replating in fresh Matrigel. The organoids were maintained in Advanced DMEM (Gibco) supplemented with 1× B27 supplement (Gibco), 1.25 mmol L^−1^ *N*‐acetyl‐l‐cysteine (Sigma–Aldrich), 10 mmol L^−1^ nicotinamide (Sigma–Aldrich), 50 ng mL^−1^ human EGF (PeproTech), 500 nmol L^−1^ A83‐01, 10 ng mL^−1^ human FGF10 (PeproTech), 5 ng mL^−1^ human FGF2 (PeproTech), 1 µmol L^−1^ prostaglandin E2 (Tocris Bioscience), 3 µmol L^−1^ CHIR 99021 (Sigma–Aldrich), 1 µmol L^−1^ forskolin (R&D Systems), 4% R‐spondin, and 4% Noggin (R&D Systems).

### TIL Culture

Briefly, surgical tumor specimens (primary tumors or lymph node metastases) obtained from tumors were minced into fragments (1–8 mm^3^) and placed into a Grex‐100 (Wilson Wolf Manufacturing) for pre‐rapid expansion phase (REP) culture. At the REP, the TILs were initially stimulated with 30 ng mL^−1^ anti‐CD3 antibody (OKT3; T&L Biotechnology) for 24 h, followed by the addition of feeders (irradiated PBMCs, at 200‐fold more than the number of TILs). At both stages, the TILs were maintained in T‐cell medium supplemented with 3000 IU L^−1^ recombinant human IL‐2 (Sihuan Pharm).

### Co‐Culture of TIL, Sell^hi^ Neutrophils, HNSCC Organoids, and Drug Treatments

To assess the antitumor effects of C‐P in vitro, coculture assays was performed involving HNSCC organoids and CD8^+^ TILs. CD8^+^ TILs were isolated and activated as described previously. On the day of the coculture, organoids were extracted from the Matrigel using cold DMEM/F12, then centrifuged at 400× g for 5 min at 4 °C. The organoids were digested into small cell clusters (30–50 µm) using TrypLE Express and resuspended in organoid culture medium. Activated CD8^+^ TILs (defined as CD45+/ CD8+) and blood‐derived Sell^hi^ neutrophils (defined as CD45+/CD15+/Sell+) and collected and cocultured with organoids at an effector‐to‐target ratio of 1:1:1 (TIL: neutrophil: tumor cell), embedded in 50% Matrigel domes. Cocultures were maintained for 72 h in a 96‐well plate (Nest Biotechnology), using a 1:1 mixture of organoid culture medium and T cell culture medium. In the experimental condition, pembrolizumab (20 µg mL^−1^, Selleck) was added, a CDK4/6 inhibitor palbociclib (1 µm, Selleck), anti‐IL15 antibody (20 ng mL^−1^, Selleck), and anti‐Stat5 antibody (1 µm, Selleck). To quantify live cell areas, 0.5 µm calcein AM and 1 µg mL^−1^ PI were added to each well and incubated for 1 h at 37 °C. After washing once with HBSS, images were captured using an EVOS M7000 system. The area of calcein AM+ and PI+ organoids was measured using ImageJ. Relative cell viability was calculated in the experimental groups using the formula [experimental calcein AM+ / control calcein AM^+^] % and normalized the values to those from the control group.

### Isolation and Sorting of Sell(hi) and Sell(lo) Neutrophils

Neutrophils were isolated from C57BL/6 bone marrow using a Neutrophil Isolation Kit (Miltenyi Biotec) according to the manufacturer's instructions. After isolation, cells were collected, centrifuged, and washed twice with PBS. Sorting of the cells was performed using a CytoFLEX flow cytometer (Beckman) based on the gating strategy for Sell^(hi)^ and Sell^(lo)^ neutrophils. Sell^(hi)^ and Sell^(lo)^ populations were defined as CD45+/CD11b+/Ly6C‐Ly6G+Sell+ and CD45+/CD11b+/Ly6C‐Ly6G+Sell‐, respectively (Figure S13, Supporting Information). FlowJo software (v.10.8.1) was used to analyze the data. Post‐sort purity results are shown in Figure S9 (Supporting Information).

### Neutrophil‐OT‐I‐T Cell‐Tumor‐OVA Co‐Culture System

MOC2‐GFP‐OVA cells (100 000 cells per well) were seeded in a 24‐well plate and cocultured with activated OT‐I CD8^+^ T cells and either Sell^(lo)^ neutrophils (CD45+/CD11b+/Ly6C‐Ly6G+Sell‐) or Sell^(hi)^ neutrophils (CD45+/CD11b+/Ly6C‐Ly6G+Sell+) at a 1:1:1 ratio for 48 h. Following coculture, both adherent and suspended cells were collected, and the killing effect of CD8^+^ T cells was assessed by flow cytometry. The specific killing percentage was calculated by comparing the number of GFP^+^ cells in the experimental group to that in the control group. In the drug treatment experiments, anti‐IL15 antibody (20 ng mL^−1^, Selleck) and anti‐Stat5 antibody (1 µm, Selleck) were added. The experimental procedure is depicted in the schematic illustration (Figure 6d).

### Flow Cytometry Analysis

For fluorescence‐activated cell sorting (FACS) analysis, single‐cell suspensions from tumor tissue, spleen, or bone marrow were first incubated with anti‐mouse CD16/CD32 antibody (Mouse Fc Block, 1:200, Biolegend) to block non‐specific binding. After blocking, cells were stained with fluorescently labeled antibodies on ice for 30 min. To distinguish live from dead cells, either Fixable Viability Dye (FITC, 1:1000, BD Biosciences) or Zombie dye (1:1000, Biolegend) was used. For intracellular staining, cells were permeabilized following surface staining using the Transcription Factor Buffer Set (BD Biosciences), and then incubated with intracellular antibodies for 60 min at room temperature. To assess IFNγ production upon restimulation, cells were stimulated with PMA (500 ng mL^−1^) and ionomycin (50 ng mL^−1^) in the presence of brefeldin A (BioLegend) for 4 h at 37 °C. The antibodies used in this study are listed in Table S14 (Supporting Information). Flow cytometry data were collected using an LSR Fortessa (BD Biosciences) and analyzed with FlowJo software (v10.8.1). FMO controls, unstained controls, and single‐staining controls were performed to ensure proper gating.

In Figure 1, analysis of epithelial, T, and NK cells was following the gating strategy shown in Figure S10 (Supporting Information). To differentiate between Ttex and Tpex, a combination of SLAMF6 and TIM‐3 markers was used. Progenitor Tex cells (Tpex) were defined as CD45+CD8+PD‐1+LY108+TIM‐3‐, while terminal Tex cells (Ttex) were identified as CD45+CD8+PD‐1+LY108‐TIM‐3+ (Figure S11, Supporting Information). For the analysis of donor OT‐I cells, STAT5+ progenitor CD8^+^ Tex cells (STAT5+Tpex) from OT‐I CD8^+^ T cells were identified using the following surface markers: CD45+CD45.1+PD‐1+LY108+TIM‐3‐STAT5+ (Figure S12, Supporting Information). In the additional target analysis, immune cells were identified based on the following surface markers: activated NK cells (CD45+NK1.1+CD107+), dendritic cells (DCs) (CD45+CD11c+MHCII+), M1 macrophages (CD45+CD11b+CD11c‐CD80+MHCII+), and M2 macrophages (CD45+CD11b+CD11c‐CD206+MHCII+).

### Adoptive Transfer of Neutrophils and OT‐I CD8 T Cell Experiments

MOC2‐OVA cells (5 × 10^4^) were orthotopically injected into C57BL/6 mice (male, 6–8 weeks old). Sell^(hi)^ and Sell^(lo)^ neutrophils were isolated from the bone marrow of MOC2 tumor‐bearing mice, as described previously, and cultured overnight. After incubation, cells were collected, centrifuged, and washed twice with PBS. Sell^(hi)^ and Sell^(lo)^ neutrophils were then sorted using a flow cytometer according to the previously outlined procedure. The experimental setup is detailed in the schematic illustrations (Figure 6h). Sell^(hi)^ and Sell^(lo)^ neutrophils (1 × 10^6^ cells per mouse) were intravenously injected into mice with orthotopic MOC2‐OVA tumors (*n* = 5 mice per group). After 12 h, the mice were intravenously injected with either activated OT‐I T cells (3 × 10^6^ cells) or control cells. The adoptive transfer of Sell^(hi)^ or Sell^(lo)^ neutrophils was repeated three times. In some experiments, the cells were labeled with Live Cell Labeling ‐ Red Fluorescence (Cytopainter, Abcam) before the final injection, following the manufacturer's instructions. The experiment was terminated when the tumors reached the endpoint. Tumors were harvested, and their volume was measured using the formula: Length × Width × Width × 0.5. Flow cytometry analysis was performed on the tumor samples.

### Bulk DNA Isolation, Library Construction, Sequencing, and Analysis

Bulk DNA isolation, library preparation, and sequencing were conducted at Sequanta Technologies Co., Ltd. in Shanghai, China. PDC libraries were prepared using a SureSelect Human All Exon V6 Capture Kit (Agilent Technologies). DNA library quality was assessed using a Qubit 3.0 fluorometer and Agilent 2100 bioanalyzer. Whole‐exome sequencing (WES) was performed on an Illumina NovaSeq 6000 system, utilizing 150‐base pair paired‐end sequencing. The average sequencing depth for PDCs was 200×. Sequence data were aligned to the human genome (GRCh38) using Burrows‐Wheeler Aligner (61), and the resulting files were converted into Binary Alignment/Map (BAM) format for downstream analysis.

### Somatic Mutation

Somatic single‐nucleotide variants (SNVs) and insertions/deletions (InDels) were identified for each paired sample using Sentieon TNseq. Variants in low‐complexity regions, such as tandem repeats and highly homologous areas, were excluded. Low‐confidence variants were filtered out if they failed to meet the following criteria: total depth > 10, alternative allele depth > 3, and mutation frequency > 0.01. High‐confidence mutations were annotated using ANOVA (Version 2016‐02‐01).

### CNV Detection

Somatic copy number variations (CNVs) were analyzed using a CNV kit by comparing normalized PDC and normal samples. CNVs were called based on an absolute log2 copy number ratio of ≥ 0.58 (log2(1.5)), which were considered as copy number losses or gains.

### Bulk RNA Isolation, Library Construction, Sequencing, and Analysis

Total RNA was extracted from cell models using TRIzol Reagent (Invitrogen), following the manufacturer's protocol, at Sequanta Technologies Co., Ltd. in Shanghai. RNA integrity was assessed using the 4200 Bioanalyzer (Agilent), and RNA concentration was quantified with the NanoDrop (Thermo Scientific). RNA purification, reverse transcription, library preparation, and sequencing were carried out at Sequanta Technologies in Shanghai, adhering to the manufacturer's instructions. mRNA was isolated from eukaryotic cells by enriching with Oligo(dT) beads following total RNA extraction. The enriched mRNA was fragmented into small pieces using fragmentation buffer and then reverse transcribed into cDNA using a NEBNext Ultra RNA Library Prep Kit for Illumina (New England Biolabs, Cat#. 7530). After repairing the ends of the purified double‐stranded cDNA, a base was added before ligating the fragments to Illumina sequencing adapters for subsequent analysis.

Ligation products were purified using AMPure XP Beads (1.0×). Fragment size was estimated via agarose gel electrophoresis, and PCR amplification was performed. After library construction, the concentration of the resulting libraries was quantified using the Qubit 3.0 fluorometer dsDNA HS Assay (Thermo Fisher Scientific), and size distribution was analyzed with the Agilent BioAnalyzer (Agilent). Sequencing was performed on an Illumina Novaseq 6000 system following the manufacturer's protocols for 2 × 150 paired‐end sequencing at Sequanta Technologies Co., Ltd. Raw reads were trimmed using Skewer (v0.2.2) to remove adapter sequences, then aligned to the reference human genome (GRCh37/hg19) using STAR (v2.4.2a). To address batch effects across datasets, the combat function was applied in the sva^[^ ^45^ ^]^ R package for batch correction. Gene expression abundance was quantified using RSEM (v1.2.29) based on uniquely mapped reads to specific regions of the human genome.

### TCGA and GEO Data Analysis

RNA‐seq expression data and corresponding clinical information for head and neck squamous cell carcinoma (HNSCC) were retrieved from the TCGA database (https://portal.gdc.com). Gene expression levels were normalized to TPM for subsequent analysis. The Foy JP et al. cohort (accession number GSE159067) was downloaded from the GEO database. Gene set variation analysis (GSVA, v1.47.3) was performed to evaluate individual samples against the cell cycle signature gene set. Survival analysis was conducted using RNA‐seq data from both the TCGA HNSCC dataset and the GSE159067 cohort. Survival curves were generated using the Survminer (v0.4.7) and survival (v3.2‐3) R packages.

### Mass Cytometry Staining and Data Acquisition for Murine Tumors

Three pooled tumor tissues were harvested from three syngeneic subcutaneous or orthotopic tumor models at specific time points and under different treatment conditions: 1) vehicle, 2) CDK4/6i, 3) anti‐PD‐1, 4) CDK4/6i + anti‐PD‐1 (day 4 for orthotopic and day 7 for subcutaneous tumors), 5) C‐PC, 6) C‐P, and 7) P‐CP (day 11 for orthotopic and day 13 for subcutaneous tumors). Tumors were enzymatically dissociated into single‐cell suspensions using 1.5 mg mL^−1^ collagenase IV, 10 mg mL^−1^ hyaluronidase type IV, and 1 mg mL^−1^ DNase I (all from Sigma–Aldrich). After digestion, cells were washed with 1× PBS and stained with 100 µL of 250 nm cisplatin (Fluidigm) for 5 min on ice to exclude dead cells. Following a brief incubation in an Fc receptor blocking solution, cells were stained with a surface antibody cocktail for 30 min on ice. The stained cells were washed twice with FACS buffer (1 × PBS + 0.5% BSA) and fixed overnight in 200 µL of intercalation solution (Maxpar Fix and Perm Buffer containing 250 nm 191/193Ir, Fluidigm). After fixation, cells were washed sequentially with FACS buffer and perm buffer (eBioscience) before staining with an intracellular antibody cocktail for 30 min on ice. Finally, cells were washed, resuspended in deionized water, mixed with 20% EQ beads (Fluidigm), and analyzed using a mass cytometer (Helios, Fluidigm). All antibodies used for mass cytometry staining are listed in Table S15 (Supporting Information).

### CyTOF Data Analysis

Raw data were de‐barcoded for each sample using a doublet‐filtering scheme with unique mass‐tagged barcodes. Then, it was normalized .fcs files from different batches using the bead normalization method.^[^ ^46^ ^]^ The data were manually gated in FlowJo to remove debris, dead cells, and doublets, retaining only live, single immune cells. Then, the Phenograph clustering algorithm was applied to categorize cells into distinct phenotypic clusters based on marker expression levels. Cell types were assigned to each cluster by analyzing marker expression patterns in a cluster‐versus‐marker heatmap. Then the t‐SNE dimensionality reduction algorithm was used to visualize high‐dimensional data in two dimensions, displaying the distribution of clusters, marker expression patterns, and differences among groups or sample types. A t‐test was finally performed to statistically analyze the frequency of each annotated cell population.

### Single‐Cell Sequencing

Tongue tumor tissues were collected from MOC2 syngeneic orthotopic tumor models at specific time points under various treatment conditions: 1) vehicle, 2) CDK4/6i, 3) anti‐PD‐1, 4) CDK4/6i + anti‐PD‐1 (day 4), and 5) C‐PC, 6) C‐P, 7) P‐CP (day 10). Tumors were enzymatically digested into single‐cell suspensions using 1.5 mg mL^−1^ collagenase IV, 10 mg mL^−1^ hyaluronidase type IV, and 1 mg mL^−1^ DNase I (all from Sigma–Aldrich). Cell concentration and viability were assessed using the Countess II Automated Cell Counter, ensuring viability remained above 90% (or above 70% for primary cells). The suspension was adjusted to a final concentration of 1000 cells µL^−1^.

The cell suspension was mixed with gel beads containing barcode information and an enzyme mixture, then encapsulated within microfluidic double‐cross droplets to form Gel Bead‐In‐Emulsions (GEMs). Each active GEM contained a gel bead (with pre‐made 10X primers), a single cell, and the Master Mix. Cells were lysed, and reverse transcription occurred within the GEMs, linking the 10X Barcode to the cDNA products. After GEMs were disrupted and the oil droplets broken, cDNA was used as a template for PCR amplification. Following cDNA amplification, the quality of the amplified products was evaluated based on fragment size and quantity. Once the quality was confirmed, sequencing libraries were constructed. The cDNA was fragmented to ≈200–300 base pairs, and end repair with adenine addition was performed. The fragments were then screened, followed by the attachment of P7 adaptors and sample indexing via PCR amplification. Final cDNA libraries were produced after an additional round of screening. After constructing the libraries, their quality was assessed. Once the library passed the quality check, sequencing was performed on the Illumina NovaSeq platform. Sequencing data were obtained and subsequently analyzed.

### ScRNA‐seq Data Processing, Cluster Annotation, and Data Integration

The filtered matrix was converted into a sparse matrix using the Seurat package (v4.4.0) with the parameters min.cells = 3 and min.features = 40, ensuring that each retained gene was expressed in at least three cells and each cell expressed a minimum of 40 genes. Quality control criteria (nFeature_RNA > 300 & nFeature_RNA < 4000 & percent.mt < 15 & nCount_RNA > 1000) were applied to retain only cells with 300–4000 detected genes, mitochondrial gene expression below 15%, and total RNA counts exceeding 1000. To further refine the dataset, the ‘doubletFinder_v3’ method was applied from the DoubletFinder package (v2.0.3) to filter out potential doublets.

The filtered dataset was normalized using the ‘NormalizeData’ function in Seurat to reduce technical variability. To identify the most variable and informative genes for downstream analyses, the ‘FindVariableFeatures’ function was used. Then the ‘ScaleData’ function was applied to standardize the dataset and performed principal component analysis (PCA) using the ‘RunPCA’ function. For dimensionality reduction, the ‘RunUMAP’ function was utilized. Following data integration, additional normalization and scaling, and applied batch effect correction using the ‘RunHarmony’ function from the Harmony package (v1.1.0) was performed. Then neighbor detection was conducted with the ‘FindNeighbors’ function (dim = 20) and clustering analysis using the ‘FindClusters’ function, applying a resolution of 1 for Maintype and 0.6 for Subtype. Cluster distributions were visualized using the ‘RunTSNE’ function. Cluster annotation was based on the expression profiles of signature genes. For extracted subpopulation cells, the same standardization pipeline was followed.

### Identification of Signature Genes

Established signature genes were retrieved from the CellMarker database and visualized their expression across different cell types using a Dotplot. To identify marker genes for each subpopulation, the ‘FindAllMarkers’ function was applied and displayed the results using the pheatmap package (v1.0.12).

### Feature Gene Expression Analysis

Gene expression was examined across various cell subpopulations using the ‘FeaturePlot’ function from the Seurat package. Additionally, gene expression density was visualized using the Nebulosa package (v1.12.0) to enhance resolution and clarity.

### Cell Ratio Analysis

Proportions of different cell types were calculated within each sample using the reshape2 package (v1.4.4) and generated bar plots with the ‘ggplot’ function for visual representation.

### RNA Velocity

To analyze RNA velocity, velocyto's run10x command was used with the mm10_rmsk.gtf.gz reference annotation file to generate loom files containing spliced and unspliced read data. Then these loom files were integrated with the single‐cell RNA‐seq dataset. RNA velocity analysis was conducted using the scVelo package (version 0.3.2), which infers cellular developmental trajectories by comparing spliced and unspliced RNA reads. Before analysis, the scv.pp.filter_and_normalize function was applied to filter and normalize the data, ensuring high quality and reproducibility. The “stochastic” model was employed to compute RNA velocity, with data preprocessing performed using the scv.pp.moments function. Velocity vectors for each cell were estimated using the scv.tl.velocity function, and all significance thresholds followed the algorithm's default settings. RNA velocity was visualized with the scv.pl.velocity_embedding_stream function, projecting the velocity vectors onto t‐SNE embeddings to illustrate the dynamic progression and directional development of CD8^+^ T cell subpopulations. Additionally, pseudotime‐based scatter plots were generated using the scv.pl.scatter function, with gnuplot color mapping applied to highlight the temporal evolution of the cells.

### Gene Ontology Analysis

Gene Ontology (GO) analysis was performed on the C‐PC and C‐P T cell clusters using the enrichGO function from the clusterProfiler package (v4.10.0). The results were visualized with a bubble plot generated using the ggplot2 package (v3.4.4).

### GSVA Analysis

Enrichment scoring was carried out using the GSVA package (v1.46.0) with reference gene sets derived from previous studies.^[^ ^3^, ^42^ ^]^ These reference gene sets include: T Cell‐Mediated Immune Response to Tumor Cells, Effector Signature, Progenitor Exhausted CD8^+^ T Cell Signature,

GSE44649_NAIVE_VS_ACTIVATED_CD8_TCELL_DN, GSE15324_NAIVE_VS_ACTIVATED_CD8_TCELL_DN, GSE9650_EFFECTOR_VS_EXHAUSTED_CD8_TCELL_UP, GSE41867_DAY8_EFFECTOR_VS_DAY30_EXHAUSTED_CD8_TCELL_LCMV_CLONE13_UP, and the ISG gene signature (Gungabeesoon et al).

Additionally, gene sets associated with angiogenesis, ECM remodeling, immunosuppression, tumor proliferation, and myeloid cell recruitment were sourced from Engblom et al.

### Differential Gene Expression Analysis

The FindMarkers function was used to analyze differential gene expression and visualized the most significantly expressed genes with a volcano plot, generated using the ggplot2 package (v3.4.4).

### Histology and Immunohistochemistry

Patient tissue and mouse tongue samples were embedded in paraffin, and 4‐µm‐thick paraffin sections were generated. Tissue morphology was assessed using hematoxylin and eosin (H and E) staining. For immunohistochemistry (IHC) assays, paraffin sections were dewaxed and rehydrated through a graded ethanol series. The sections underwent heat‐mediated antigen retrieval with Citrate Unmasking Solution (Cell Signaling Technology, CST) and were blocked with goat serum (Beyotime) at room temperature for 1 h. Primary antibodies, diluted in 3% BSA (Beyotime), were incubated overnight at 4 °C. The color reaction was developed using DAB (CST). All H and E and IHC images were captured with an OLYMPUS BX51 microscope and a DP 71 camera (OLYMPUS).

A pathologist (manual visual assessment, AS) scored the samples based on the intensity of Cyclin D1 and pRb807/811 tumor epithelial staining. Scores ranged from 0 to 4, with an associated prevalence score (by area), yielding an overall H‐score calculated as follows: (H1 ^*^ area%) + (H2 ^*^ area) + (H3 ^*^ area). Samples were excluded from analysis if staining quality was poor (e.g., technical staining failure) or if insufficient tumor epithelium was present on the slide. CD3^+^, CD4^+^, and CD8^+^ cells were counted in five random high‐power fields (1 mm^2^) at the tumor border and five in the tumor center. The average count for each sample was calculated. Programmed cell death‐ligand 1 (PD‐L1) expression was evaluated using the PD‐L1 IHC 22C3 pharmDx assay (Dako). The combined positive score (CPS) was defined as the total number of PD‐L1‐stained cells (including tumor cells, tumor‐associated lymphocytes, and macrophages) divided by the total number of viable tumor cells, multiplied by 100.

### Multiplex Immunofluorescence Analysis

Multiplex immunofluorescence (IF) staining was conducted using TSA 6‐plex and 7‐plex Kits (Servicebio) following the manufacturer's instructions. Different primary antibodies were applied sequentially, followed by incubation with horseradish peroxidase‐conjugated secondary antibodies and tyramide signal amplification working solution. After each tyramide signal amplification step, the slides were microwave heat‐treated. Multiplex IF for three panels was performed, each containing distinct primary antibodies: Panel 1: Pan‐CK (clone AE1/AE3, dilution 1:1, Dako), CD8 (1:200, abcam), PD‐L1 (1:200, abcam), phospho‐Rb (1:800, CST), Cyclin D1 (1:500, CST), and CD3 (1:500, abcam); Panel 2: CD4 (1:500, abcam), CD8 (1:200, abcam), PD‐1 (5 µg mL^−1^, abcam), Ly6G (1:200, CST), and CK5 (1:200, CST). Panel 3: CK5(1:200, CST), α‐SMA (1:500, ServiceBio), CD3(1:500, abcam), IL15 (1:200, preprotech); Panel 4: CD11c (1:200, ServiceBio), F4/80 (1:500, ServiceBio), Ly6G (1:200, CST), IL15 (1:200, preprotech). Nuclei were stained with DAPI (Sigma–Aldrich) after labeling all primary antigens. For each patient and mouse tumor sample, slides were scanned using the Pannoramic MIDI (3DHISTECH), capturing five fields of view for multispectral imaging analysis based on tissue size. A pathologist reviewed the selected fields, and quantitative analysis was performed using the inForm image analysis software (v.2.4).

### Protein Extraction and Immunoblotting

OT‐I CD8^+^ T cells were washed twice with ice‐cold 1x phosphate‐buffered saline (PBS; Invitrogen) and lysed using lysis buffer (Beyotime) containing 50 mm NaCl, 50 mm EDTA, and 1% Triton X‐100, supplemented with a protease inhibitor cocktail (Selleck). Protein concentration was determined using a Pierce BCA Protein Assay Kit (Thermo). Protein lysates were then separated by sodium dodecyl sulfate‐polyacrylamide gel electrophoresis (SDS‐PAGE; Beyotime) and transferred to polyvinylidene fluoride (PVDF) membranes (Millipore). After blocking with 5% (w/v) nonfat dry milk (BD Biosciences) for 1 h, the membranes were incubated overnight at 4 °C with primary antibodies: anti‐Stat5 (1:1000, CST), anti‐phospho‐Stat5 (1:1000, CST), and anti‐GAPDH (1:1000, CST). The membranes were then washed with TBS containing 0.05% Tween (Beyotime) and incubated with horseradish peroxidase (HRP)‐conjugated secondary antibodies (1:5000, CST) for 1 h at room temperature. Protein bands were detected using SuperSignal West Femto Maximum Sensitivity Substrate (Thermo Fisher), and images were captured using the 5200‐Multi Automatic Chemiluminescence Imaging System (Tanon).

### ELISA for and IL15

Sell^(hi)^ and Sell^(lo)^ neutrophils were isolated by cell sorting and cultured in serum‐free media for 24 h. Cytokine production from each group was quantified using the Mouse IL15 DuoSet ELISA (R&D Systems) in accordance with the manufacturer's guidelines. Absorbance was measured on a Synergy Neoplate reader (BioTek) using Gen5 software.

### Statistics and Reproducibility

All statistical analyses were performed using GraphPad Prism 9.5.0 software. Statistical significance was calculated by two‐tailed unpaired t‐test on two experimental conditions or one‐way ANOVA on three or more experimental conditions, with *P* < 0.05 considered statistically significant as indicated in each figure legend. The exact P values are reported in each figure, except when *P* < 0.0001. Power analyses were used to predetermine sample sizes. The exact sample sizes are indicated in the figure legends. Independent experiments were pooled and analyzed together whenever possible, as detailed in the figure legends. Animals were excluded only if they died, had to be killed according to protocols approved by the animal experimental committees, or when the measurement was not reliable for technical issues (specifically for orthotopic models). For in vitro experiments, no data were excluded. For in vivo studies, tumor measurement, treatment, and analysis were performed blindly by different researchers to ensure that the studies were run in a blinded manner. Animals were randomized, with each group receiving mice with similar tumor size or similar body weight. For in vitro studies, randomization and blinding of cell lines were not possible; however, all cell lines were treated identically without prior designation. All graphs show mean values ± s.e.m or s.d, as detailed in the figure legends.

## Conflict of Interest

The authors declare no conflict of interest.

## Author Contributions

Y.Z., B.S., R.Z., and Z.G. contributed equally to this work. Y.Z., B.S., R.Z., Z.Z.G., C.S., and Y.H. performed experiments; Z.Z.G., W.T., and W.W.Z. performed bioinformatic analyses; Y.Z., B.S., C.S., and Y.H. prepared experimental reagents; R.Z., W.C.Z., and L.L. helped discuss the results; J.L. and Z.Z. prepared human samples; Y.Z. analyzed data with B.S., R.Z., W.W.Z. and Z.Y.G. and W.W.Z. drafted the manuscript. Z.Z.G. conceived and designed the project with W. W.Z., Z.Z, J.L., Q.Z., and L.L.Z.

## Supporting information



Supporting Information

Supplemental Table 1

Supplemental Table 2

Supplemental Table 3

Supplemental Table 4

Supplemental Table 5

Supplemental Table 6

Supplemental Table 7

Supplemental Table 8

Supplemental Table 9

Supplemental Table 10

Supplemental Table 11

Supplemental Table 12

Supplemental Table 13

Supplemental Table 14

Supplemental Table 15

## Data Availability

The raw sequence data of humans reported in this paper have been deposited in the Genome Sequence Archive^[^ ^47^ ^]^ in the National Genomics Data Center,^[^ ^48^ ^]^ China National Center for Bioinformation / Beijing Institute of Genomics, Chinese Academy of Sciences (GSA‐Human: HRA008082) that are publicly accessible at https://ngdc.cncb.ac.cn/gsa‐human. The raw sequence data of the mouse reported in this paper have been deposited in the Genome Sequence Archive (Genomics, Proteomics and Bioinformatics 2021) in the National Genomics Data Center (Nucleic Acids Res 2022), China National Center for Bioinformation / Beijing Institute of Genomics, Chinese Academy of Sciences (GSA: CRA017953) that are publicly accessible at https://ngdc.cncb.ac.cn/gsa. The publicly available HNSCC RNA‐seq data can be accessed in the TCGA (TCGA‐HNSCC) dataset and GSE159067. Source data are provided in this article. All other data supporting the findings of this study are available from the corresponding author upon reasonable request. Custom code scripts and specific parameters used for data processing and analysis are available at https://github.com/Weiwen1807/Palbociclib_project.
