# Supplementary material for: CDK4/6 Inhibitor Priming Enhances PD‐1 Blockade via Sellhi Neutrophil‐Induced Stat5a+ Progenitor Exhausted CD8+ T Cell
Source: Adv Sci (Weinh). 2025 Sep 11;12(44):e10501. doi: 10.1002/advs.202510501 (PMC12667468; doi:10.1002/advs.202510501)

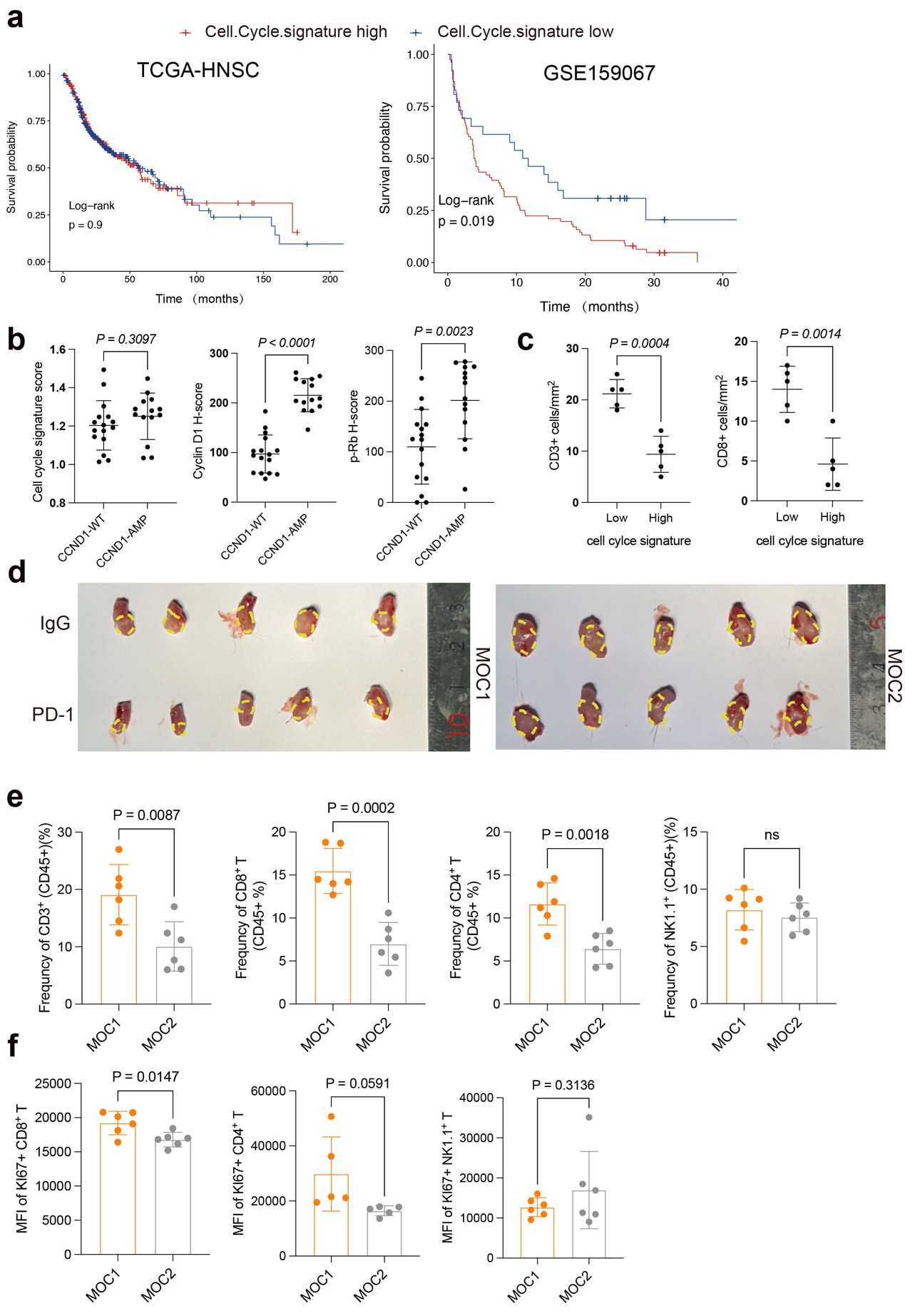


**Supplementary Fig. S1:** **Cyclin D1-CDK4/6 signaling is associated with T cell exclusion and immunotherapy resistance, Related to Fig. 1**. **a,** GSVA was performed using a reference cycle gene set for enrichment scoring in the bulk RNA data for tumor samples of HNSCC, followed by survival analysis based on the clinical information from TCGA (left panel) and GSE159067 (right panel) datasets. **b,** Comparison of cell cycle signature score, cyclin D1 and pRb expression levels between CCND1 WT and AMP groups. **c,** Comparison of CD3 and CD8^+^ T cell numbers between top 5 cell cycle high patients and top 5 cell cycle low patients. **d,** Photographs of tumors in Fig. 1d. **e,** Flow cytometry analysis of frequencies of CD3^+^, CD8^+^, CD4^+^ T cells and NK1.1^+^ NK cells in MOC1 or MOC2 tumors (n = 6). **f,** Flow cytometry of Ki-67 expressed in CD4^+^, CD8^+^, NK1.1^+^ cell in MOC1 or MOC2 tumor, n=5-6. MFI, mean fluorescence intensity. Two-sided unpaired t-test**.** Data in (**b, c, e, f**) are presented as mean ± s.d.


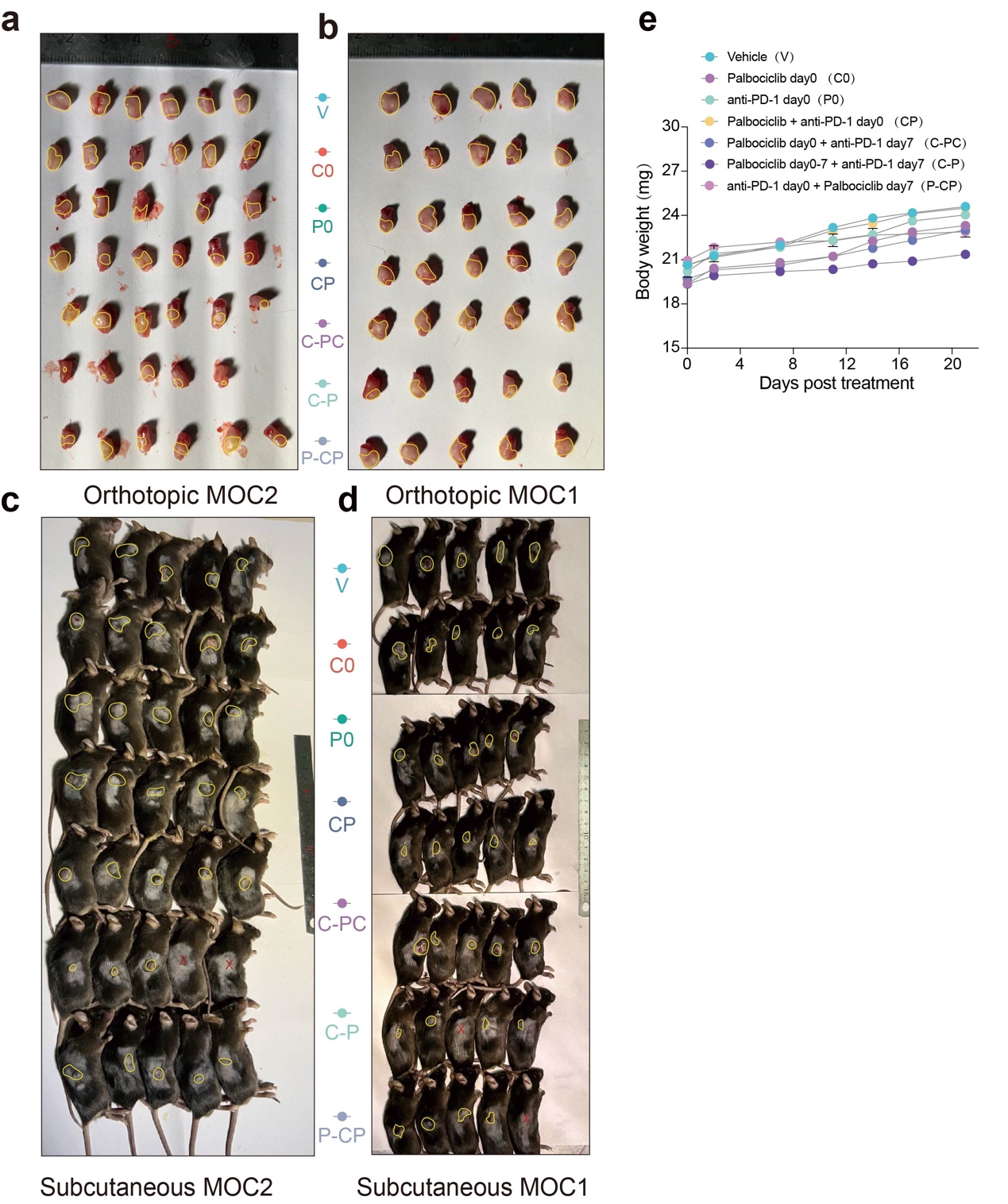


**Supplementary Fig. S2: CDK4/6i priming before PD-1 blockade shows superior antitumor activity than concurrent regimens and overcome immunotherapy resistance, related to Fig. 2.** **a-b,** Photographs of tumors in Fig. 2a. **c-d**, Photographs of tumors in Fig. 2d. **e**. Body weight of mice in each treatment group in subcutaneous MOC1 tumor bearing mice treated with indicated regimens. Data in (**h**) are presented as mean ± s.d.


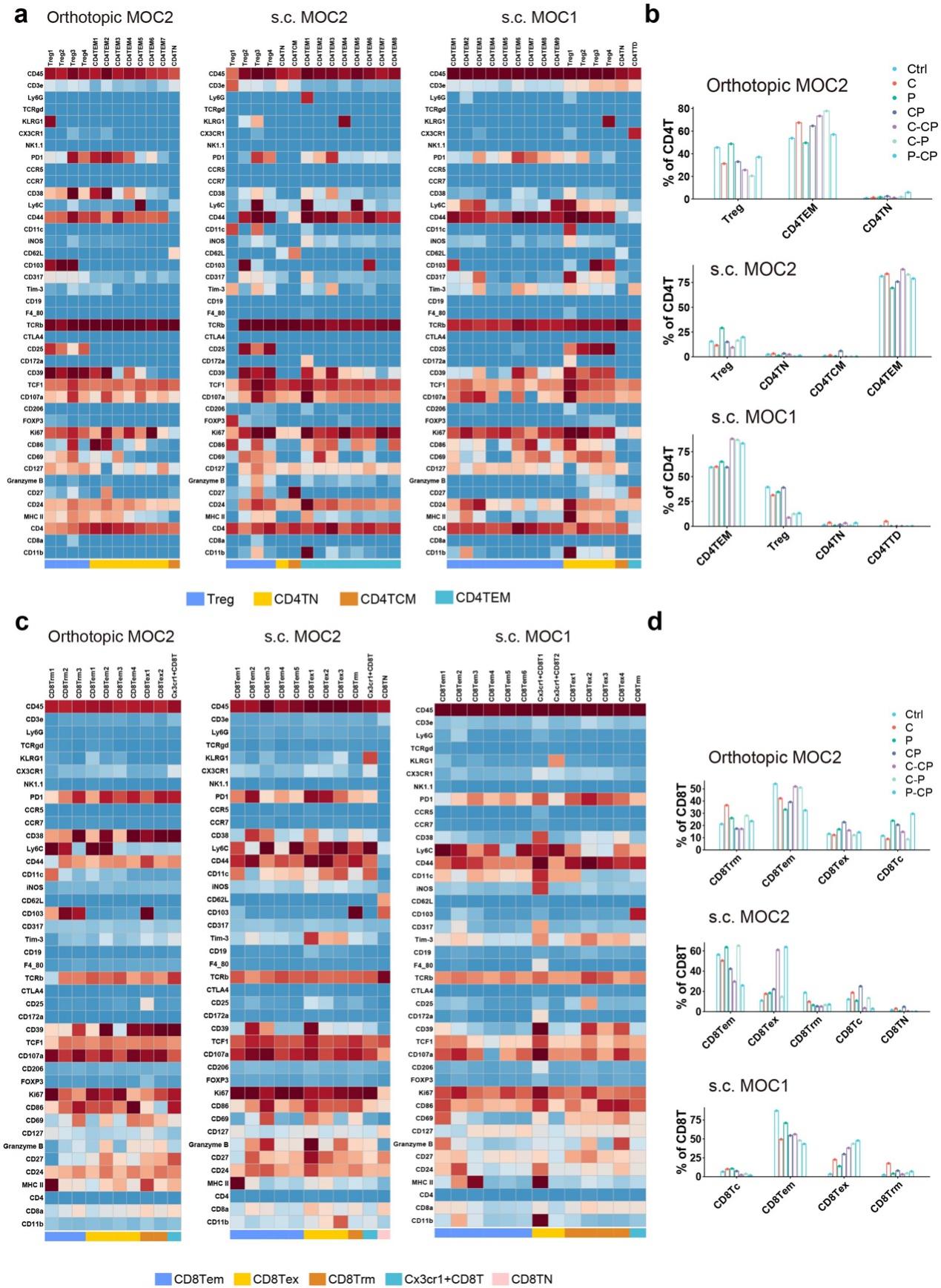


**
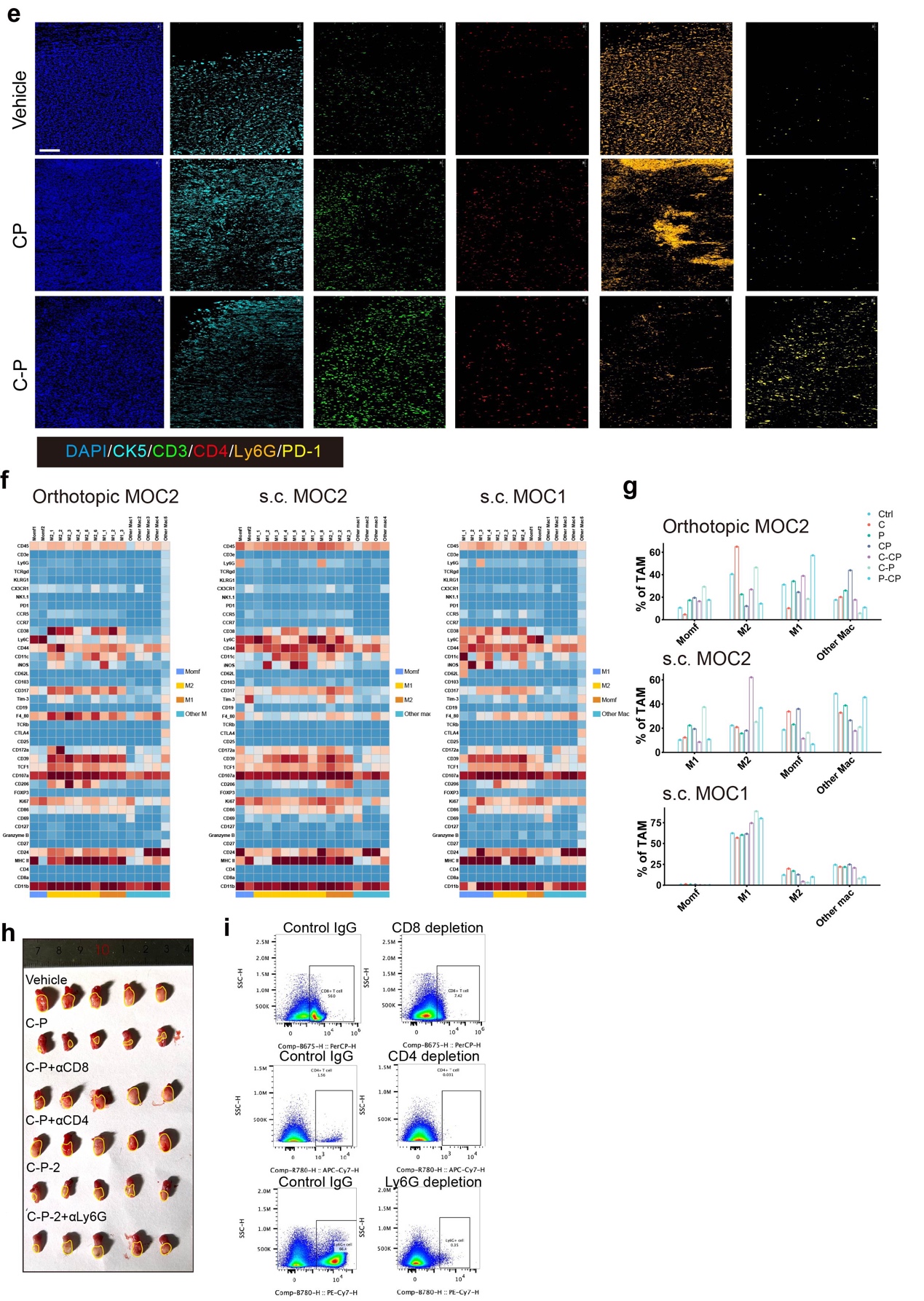
Supplementary Fig. S3: T cell expansion and M1-like TAM is associated with response to optimized therapy regimen, related to Fig. 3.** (**a-d, f-g**), Heatmaps showing the expression values of immune phenotypic protein markers across multiple subsets in tumor-infiltrating CD4^+^ (**a**), CD8^+^ (**c**), TAM (**f**) cells analyzed by CyTOF in three indicated syngeneic tumor models. The expression values of each marker were normalized to the maximum mean value across subsets. Tn (naive): CD62L+CD44-; Trm (resident memory): CD103+CD44+; Tem (effector memory): CD62L-CD44+; Tex (exhausted): PD-1+TIM3+; Tc (chemotaxis): Cx3cr1+., Frequencies of subsets of CD4^+^ (**b**), CD8^+^ (**d**) and TAM cells (**g**) in the CD45^+^ population of three syngeneic tumor models in different treatment regimens indicated. **e,** Representative images of mIF staining of CK5, Ly-6G, CD4, CD8, PD-1 in V, CP and C-P regimens of MOC2 orthotopic tumors Scale bar, 200μm. **h**, Photographs of tumors in Fig. 3g and h. **i,** Representative plots showing depletion efficiency in tumor by CD4, CD8 and Ly6G antibodies.


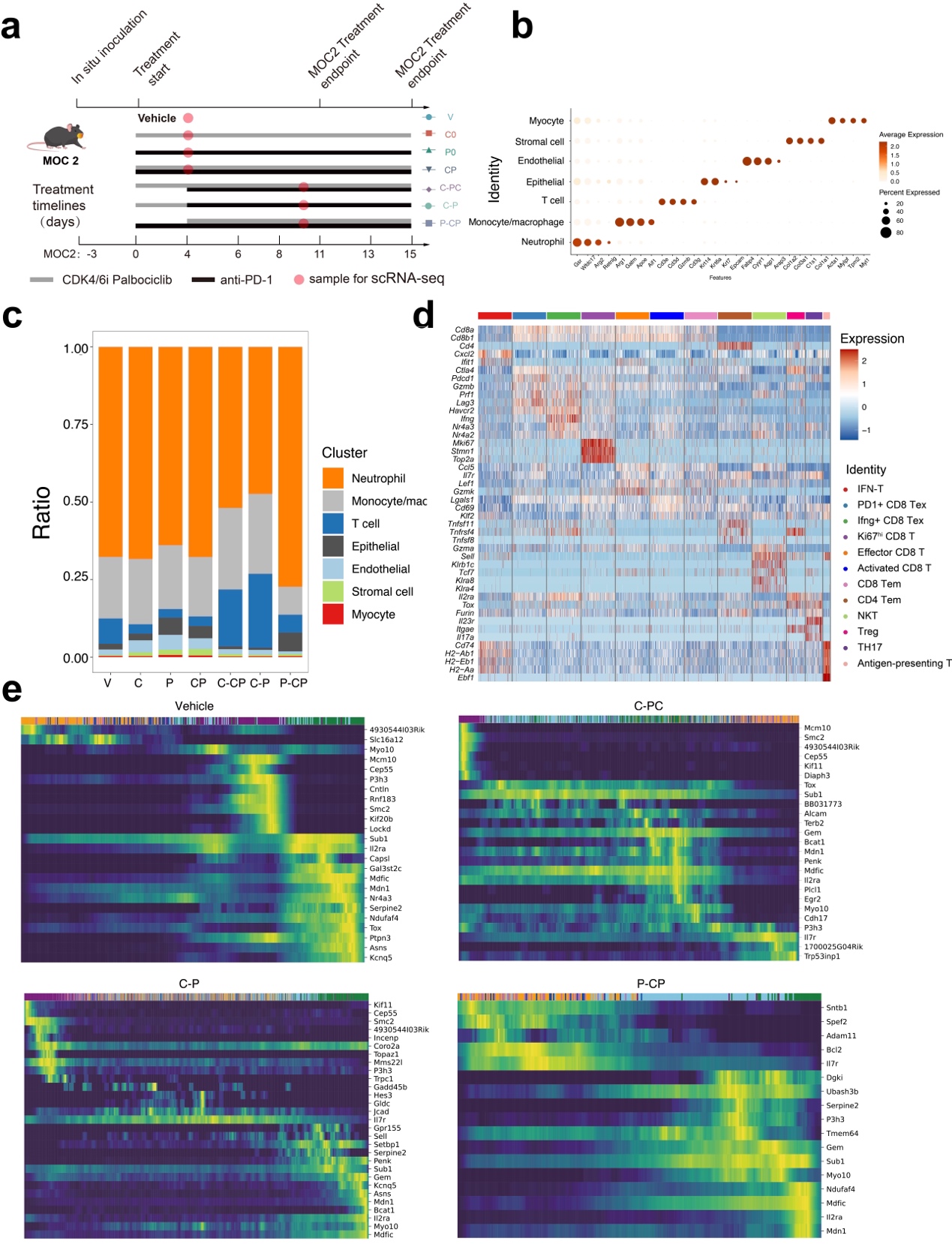


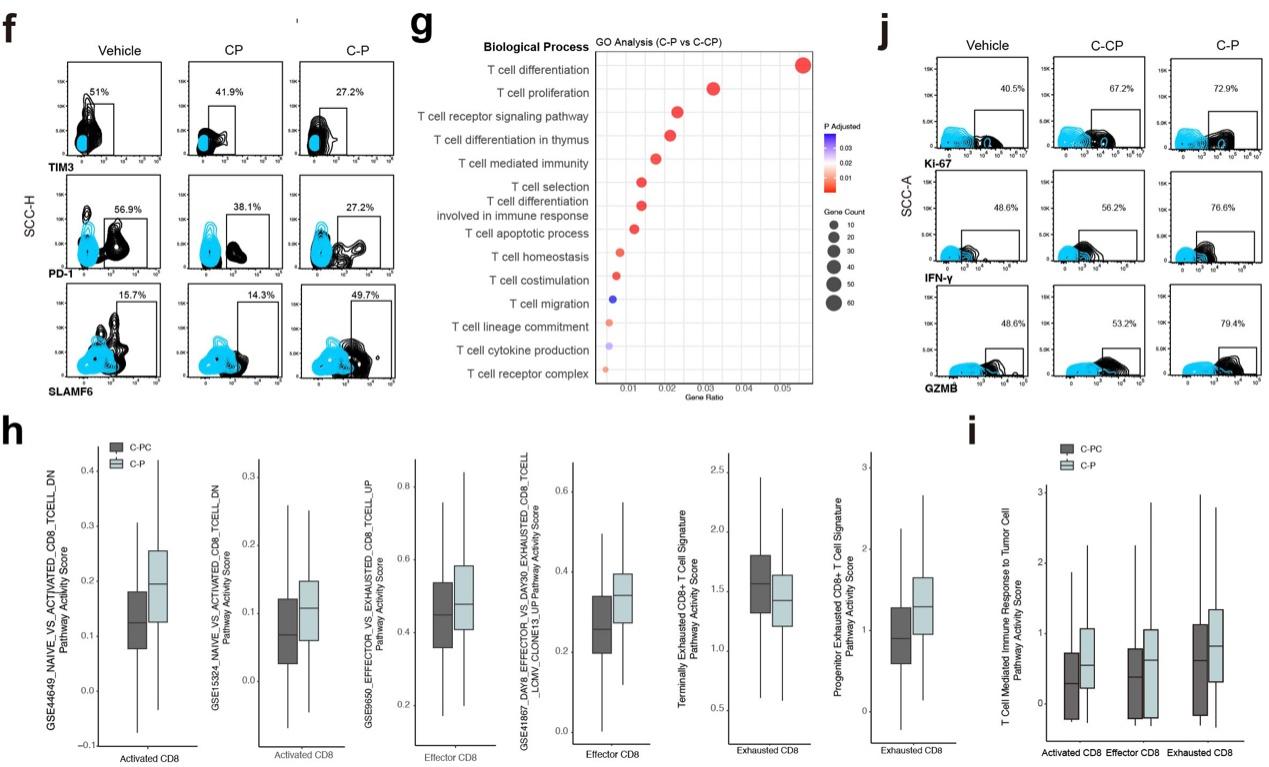


**Supplementary Fig. S4: CD8^+^ Tex was reversed to form progenitor-like Tex and retain proliferative and cytotoxic capacity under C-P regimens. Related to Fig.4 a**, Experimental design. Three pooled tongue tumor tissues were collected from MOC2 syngeneic orthotopic tumor models at each time points and in different treatment regimens including: (1) vehicle, (2) CDK4/6i, (3) anti-PD-1, (4) CDK4/6i + anti-PD-1 (on d4); (5) C-PC, (6) C-P, (7) P-CP (on d10). Red circle indicated the timepoint of sample collection for ScRNA-seq. **b**, Maintype marker gene expression was visualized using a bubble plot. **c**, Visualization of the proportion of Maintype in each sample using a bar plot. **d**,Visualization of marker gene expression for T cell subgroups using a pheatmap. **e**, Visualization of top genes from the pseudotime analysis of CD8 T cell subgroups using scVelo. **f**, Representative FACS plots of flow cytometry of PD-1, TIM3, SLAMF6 in CD8^+^ T cells from vehicle, CP, and C-P groups. **g,** GO (Gene Ontology) analysis of differentially expressed genes of T cell between C-P and C-CP. **h**, Visualization of GSVA enrichment scores for differentially expressed genes (DEGs) between the C-PC and C-P groups in various CD8 T cell clusters along indicated pathways using box plots. **i,** Visualization of GSVA enrichment scores using "T Cell Mediated Immune Response to Tumor Cell" gene set for differentially expressed genes between the C-PC and C-P groups among indicated CD8^+^ T cell clusters. **j,** Representative FACS plots of flow cytometry of Ki-67, IFNγ, Granzyme B in CD8^+^ T cells from vehicle, CP, and C-P groups.
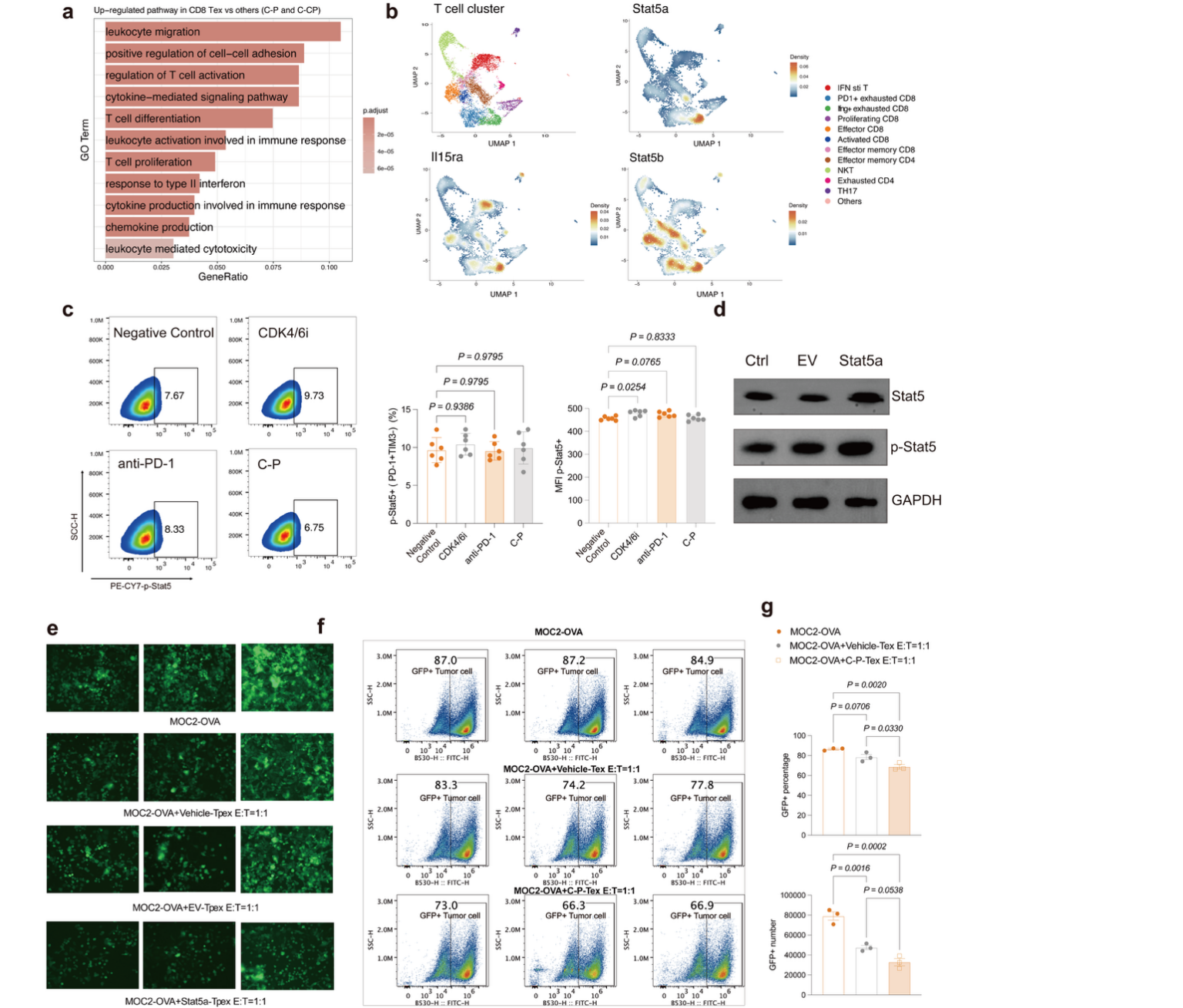


**Supplementary Fig. S5: Stat5a mediates the functionally antitumor phenotype of CD8^+^Tpex cells. Related to Figure 5. a,** Go analysis of CD8^+^ Tex and other CD8^+^ T cells in C-P and C-CP group. **b**, UMAP visualization of gene expression density for *Il15ra*, *Stat5a*, *Stat5b* in T cell subgroups. **c,** T cells isolated from spleen of WT mice were routinely cultured with IL-2 containing-medium and activated. Then, cells were treated with PBS, CDK4/6 inhibitor, PD-1 antibody, and C-P regimen and harvested for cell staining. Representative FACS plots for p-Stat5^+^ PD-1^+^ TIM3^-^ OT-I CD8+ T cell in indicated treatment groups and the frequencies and MFI are also shown, n=6, one-way ANOVA test. **d**, Total Stat5 and phospho-Stat5 expression detected by western blot on Stat5a-overexpression, empty vector OT-I CD8^+^T cells and control (untransduced wild-type OT-I CD8^+^T cells). **e,** Representative images from figure 5g. **f-g**, Cell killing of GFP+ target cells during the co-incubation of MOC2-OVA-GFP tumor cells with Tex CD8+ cells sorted from vehicle or C-P treated MOC2-OVA tumor bearing mice for 48h under indicated treatments (n=3). one-way ANOVA test. Data in (**c, g**) are presented as mean ± s.d.

**
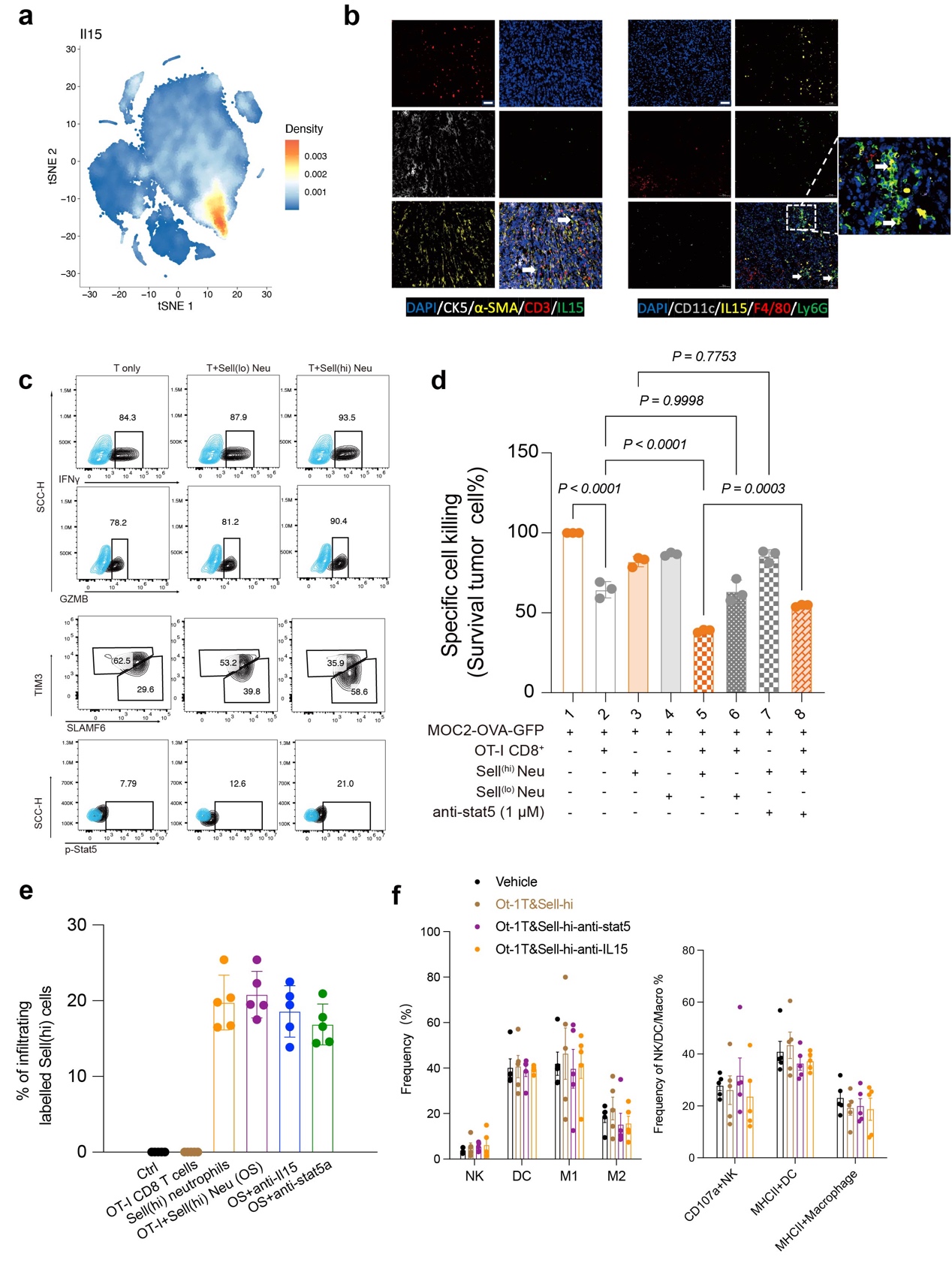
**

**
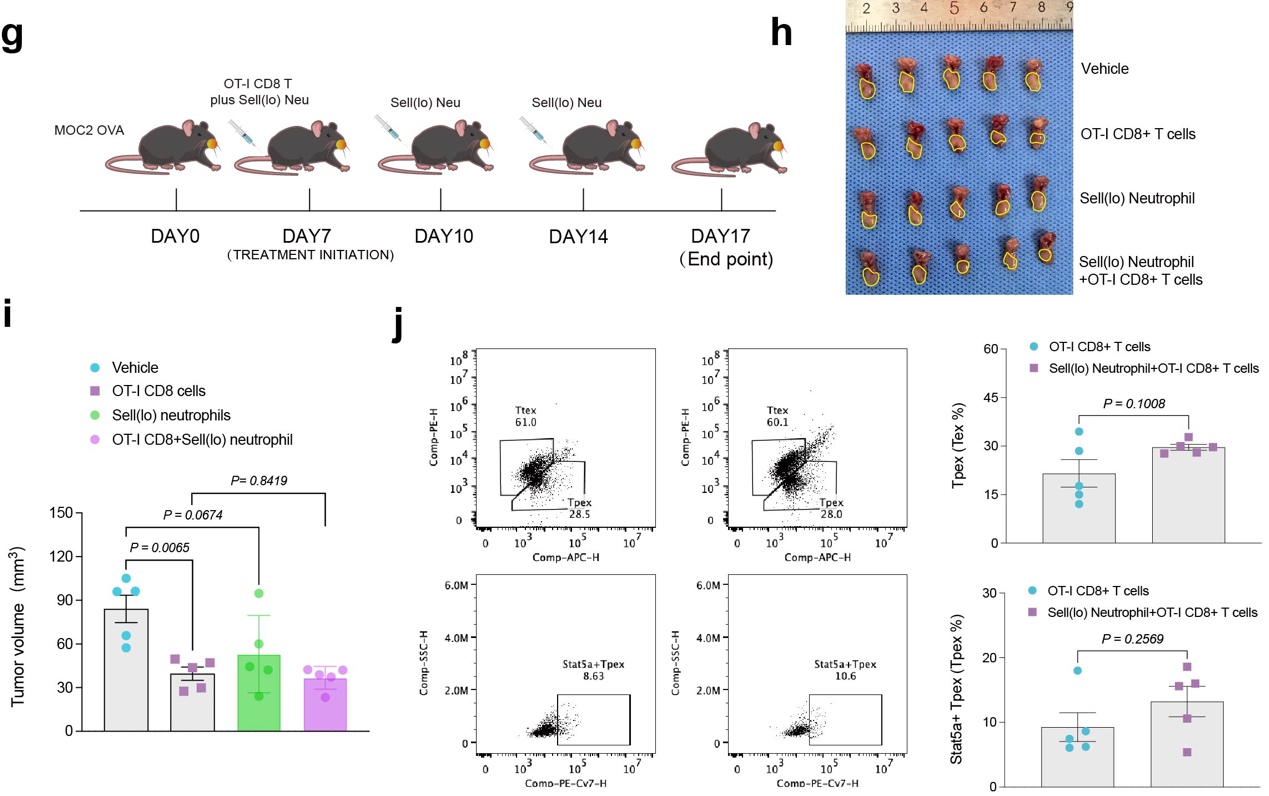
Supplementary Fig. S6:** **Interferon-stimulated neutrophils induced CD8^+^Tpex cells through IL15-Stat5a axis.** **Related to Figure 6.** **a**, tSNE visualization of gene expression density for Il15 in maintype subgroups. Related to Fig 4a. **b**, Representative images of mIF staining of Panel A: CK5, α-SMA, CD3, IL15, DAPI; Panel B: CD11c, F4/80, Ly6G, IL15, DAPI in MOC2 orthotopic tumor, scale bar = 100 μm. **c,** Representative FACS plots for Tpex, Ttex, p-Stat5^+^ Tpex, IFNγ^+^, and GZMB^+^OT-I CD8^+^ T cell in indicated treatment groups. **d**, Specific cell killing of GFP^+^ target cells during the co-incubation of MOC2-OVA-GFP, sorted Sell^(hi)^ and Sell^(lo)^ neutrophils and CD8^+^ T-cells from OT-I mice under indicated treatments and at the indicated times (n=3)，one-way ANOVA test. **e**, Frequency of Sell(hi) neutrophils that have infiltrated into the tumor, as tracked by APC cell labeling. **f**, Bar plots showing the frequency of NK cells, DCs (Dendritic Cells), M1 Macrophages, and M1 Macrophages and the proportion of CD107a+ NK cells, MHCII+ DCs, and MHCII+ Macrophages in the tumor microenvironment under different drug treatments. **g**. Schematic overview of the in vivo experiment, whereby Sell(lo) neutrophils and OT-I CD8+ T cells are adoptively transferred into mice bearing MOC2 OVA tumors. In brief, MOC2 OVA tumors were orthotopically injected into C57BL/6 mice and treatment with Sell(lo), OT-I CD8+ T cells or combination therapy, was initiated on Day 7. Additional doses were given throughout the time-course as marked. On day 17, mice were sacrificed, and tumors were collected for data analysis. **h-i**, Tongue images (h) and volume quantifications (i) of in vivo experiment using MOC2-OVA tumor orthotopic model treated with indicated treatments. Yellow circle denotes the tumor area in tongue tissue. **j**, Representative FACS plots (top) and quantification (bottom) of flow cytometry analysis of frequencies of Tpex CD8+, Stat5a+ Tpex CD8+ in OT-I CD8+ T cells and Sell(lo) neutrophils combined with OT-I CD8+ T cells groups (n = 5), Unpaired t test.


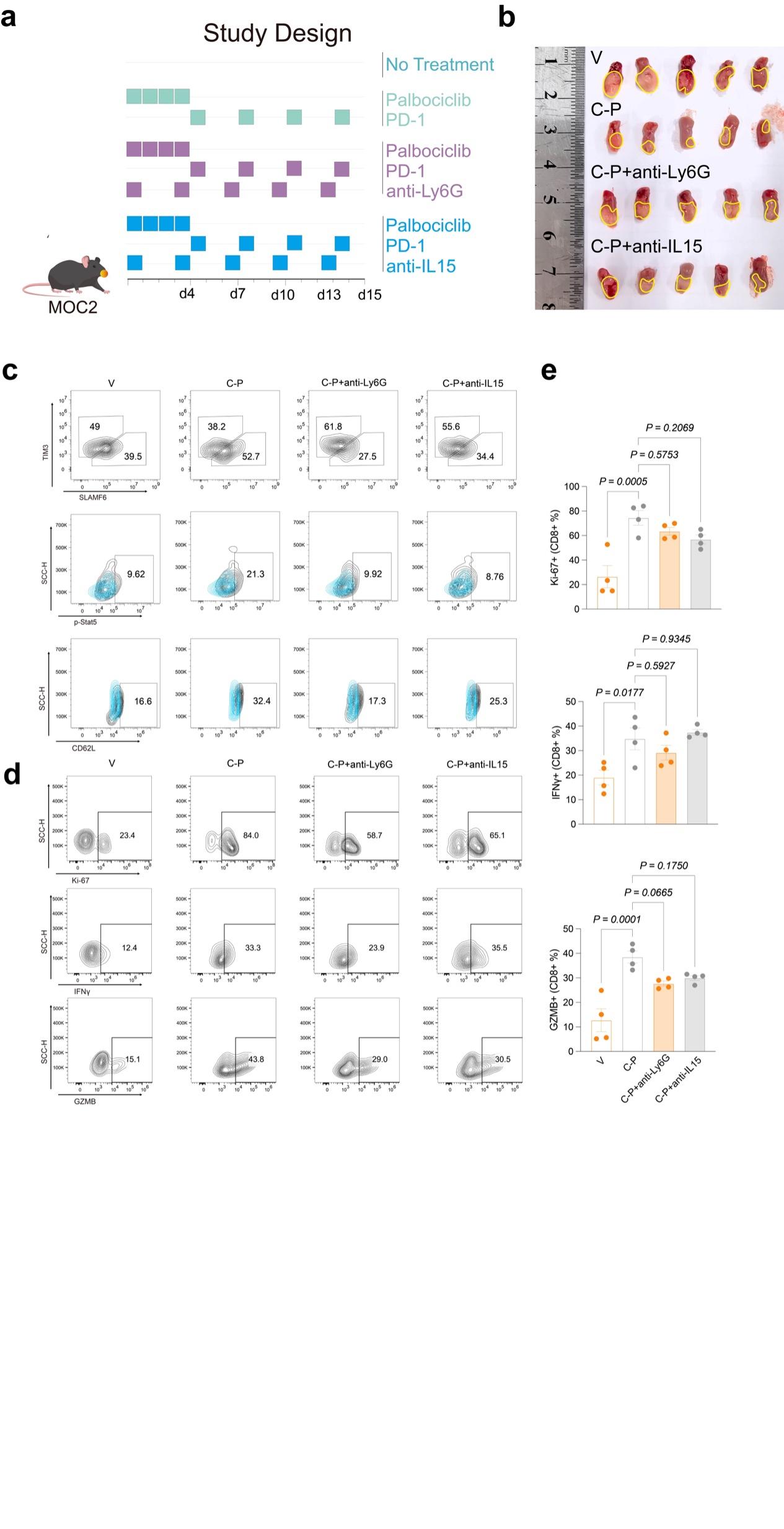


**Supplementary Fig. S7: Blocking IL15-Stat5a signaling dampens antitumor activity of C-P regimen, Related to Figure 7. a,** Experimental design. **b,** Images of tumors collected from each group at the endpoint of experiments in Figure 7a. **c,** Representative FACS plots for Tpex, Ttex, p-Stat5^+^ Tpex, and Sell^(hi)^ neutrophil in indicated treatment groups. **d-e**, Representative FACS plots for Sell^(hi)^ neutrophils and p-Stat5^+^ Tpex OT-I CD8^+^ T cell in indicated treatment groups (**d**) and frequencies are shown (**e**), one way ANOVA test. Data in (**e**) are presented as mean ± s.d.

**Supplementary Fig. S8: Schematic diagram of this study.**

**
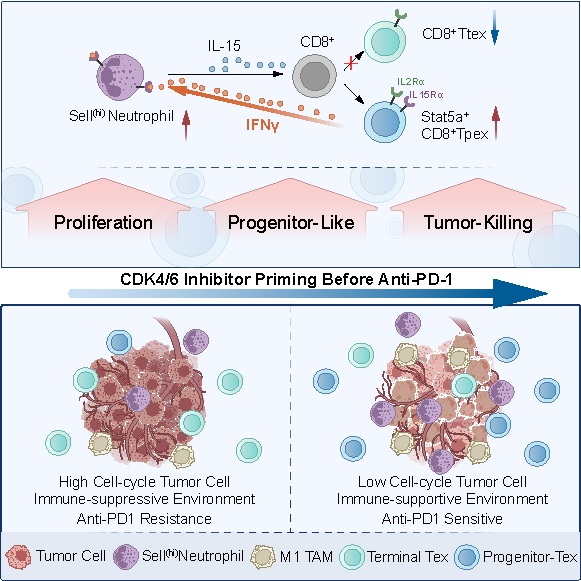
**

**Supplementary Fig. S9：**Post-sort purity check. Gating strategies for the sorted Sell(hi)and Sell(lo) neutrophils. b, Gating strategies for the stat5a and empty vector lentiviral transduced Tpex.

**
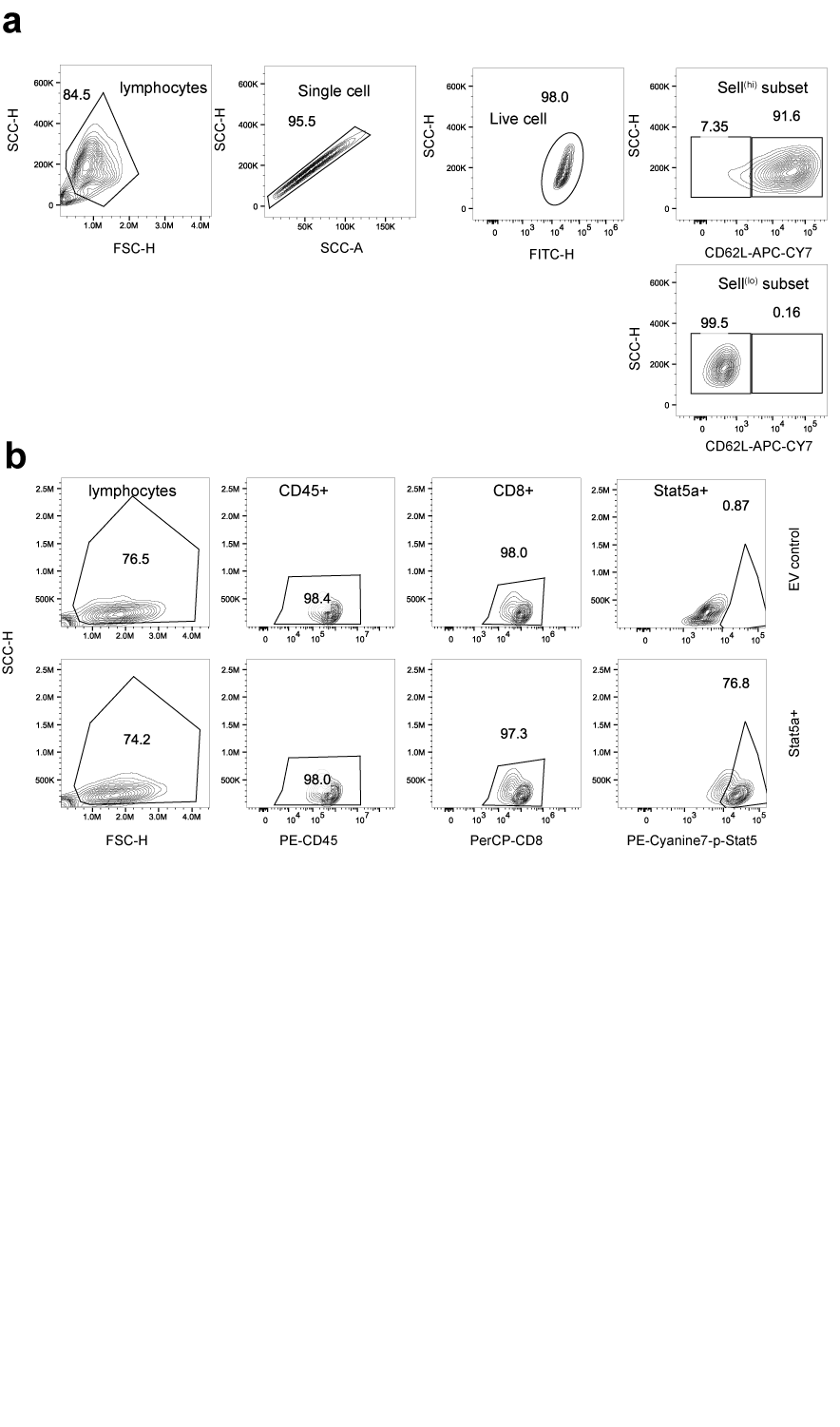
**

**Supplementary Fig. S10：**Gating strategies. a, Gating strategies for the T, NK cells. b, Gating strategies for the epithelial cells.

**
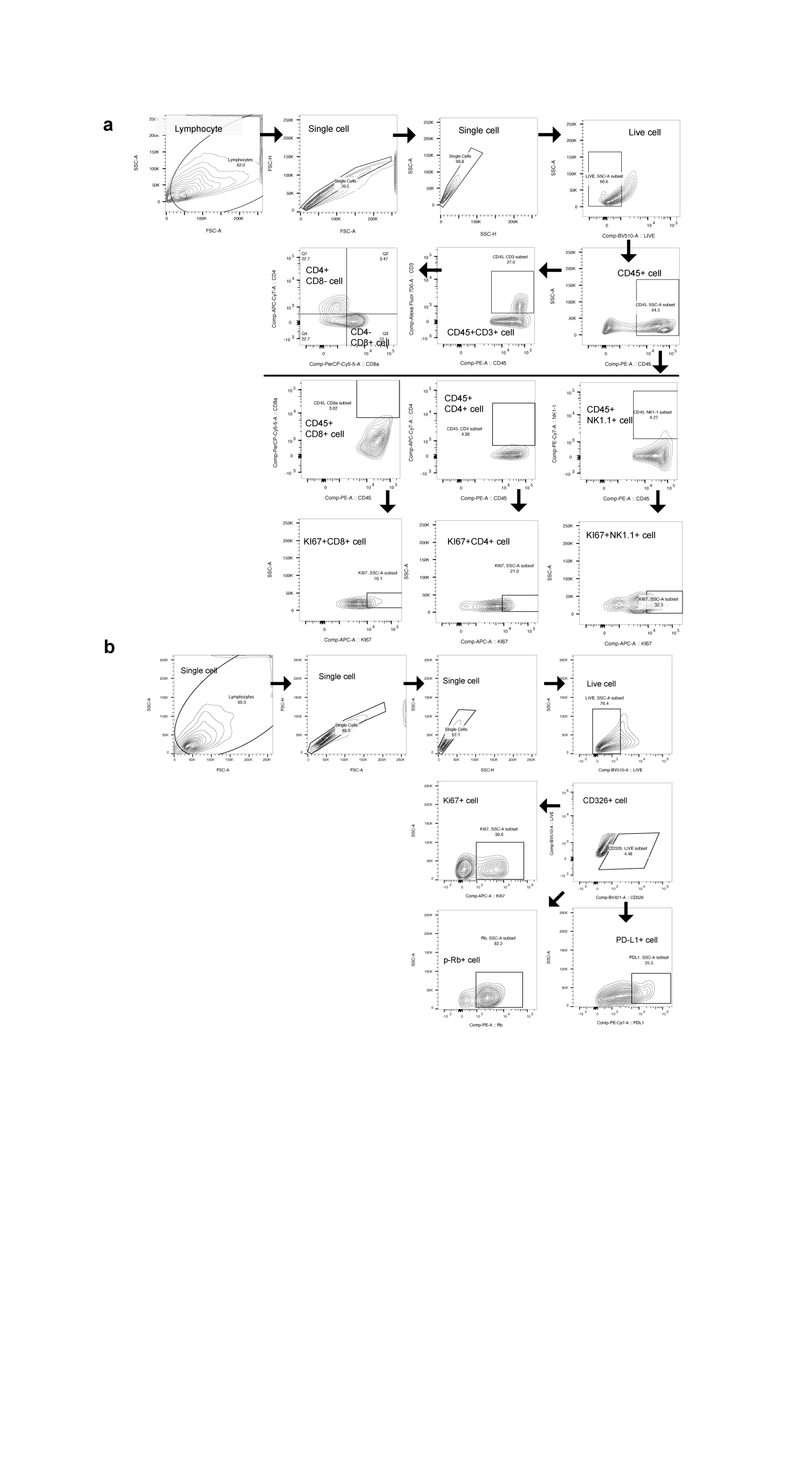
**

**Supplementary Fig. S11：**Gating strategies. a, Gating strategies for the PD-1+/TIM3+/SLAMF6+ CD8+ T cells. b, Gating strategies for the GranzymeB+/IFNγ+/Ki-67+ CD8+ T cells.


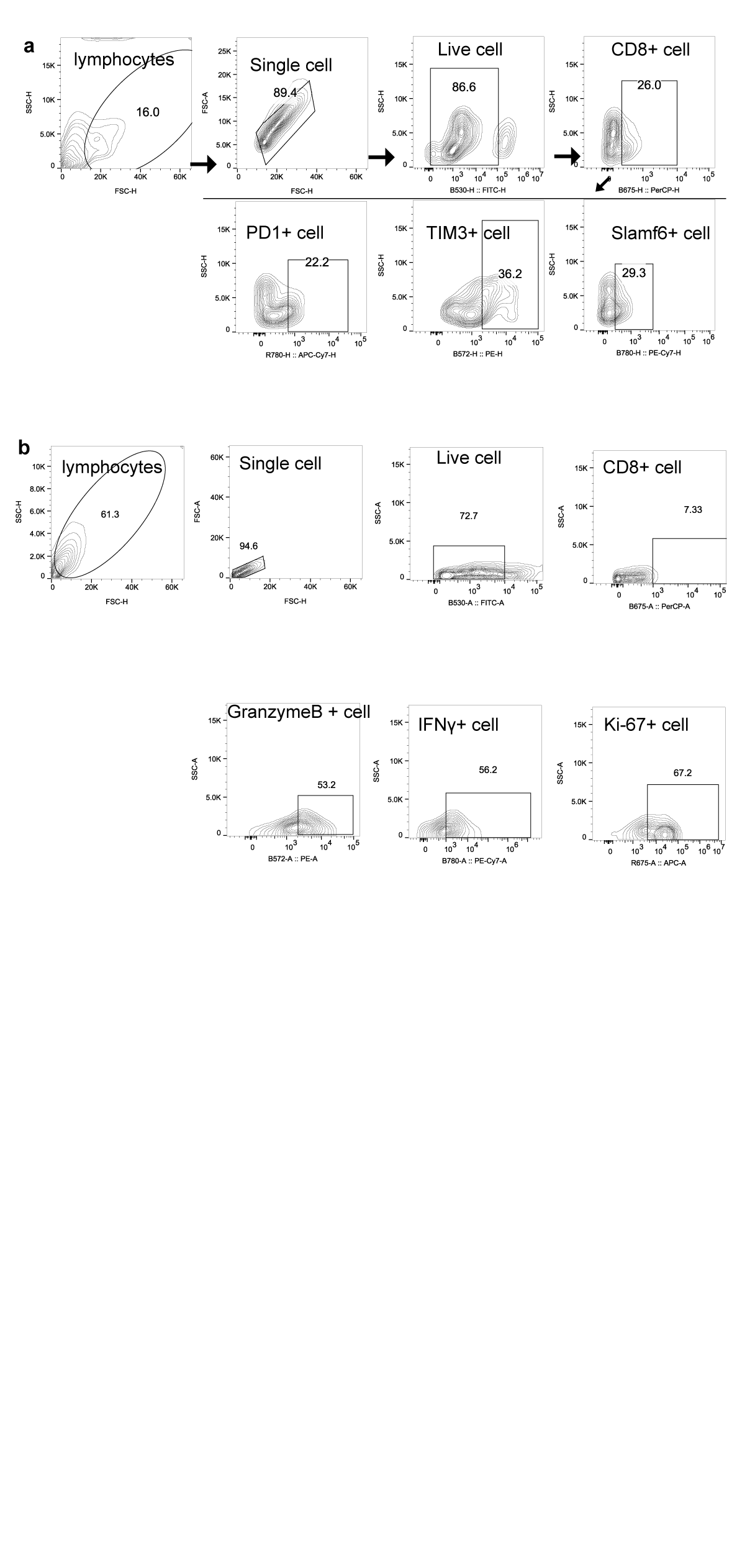


**Supplementary Fig. S12：**Gating strategies for the p-Stat5 Tpex.


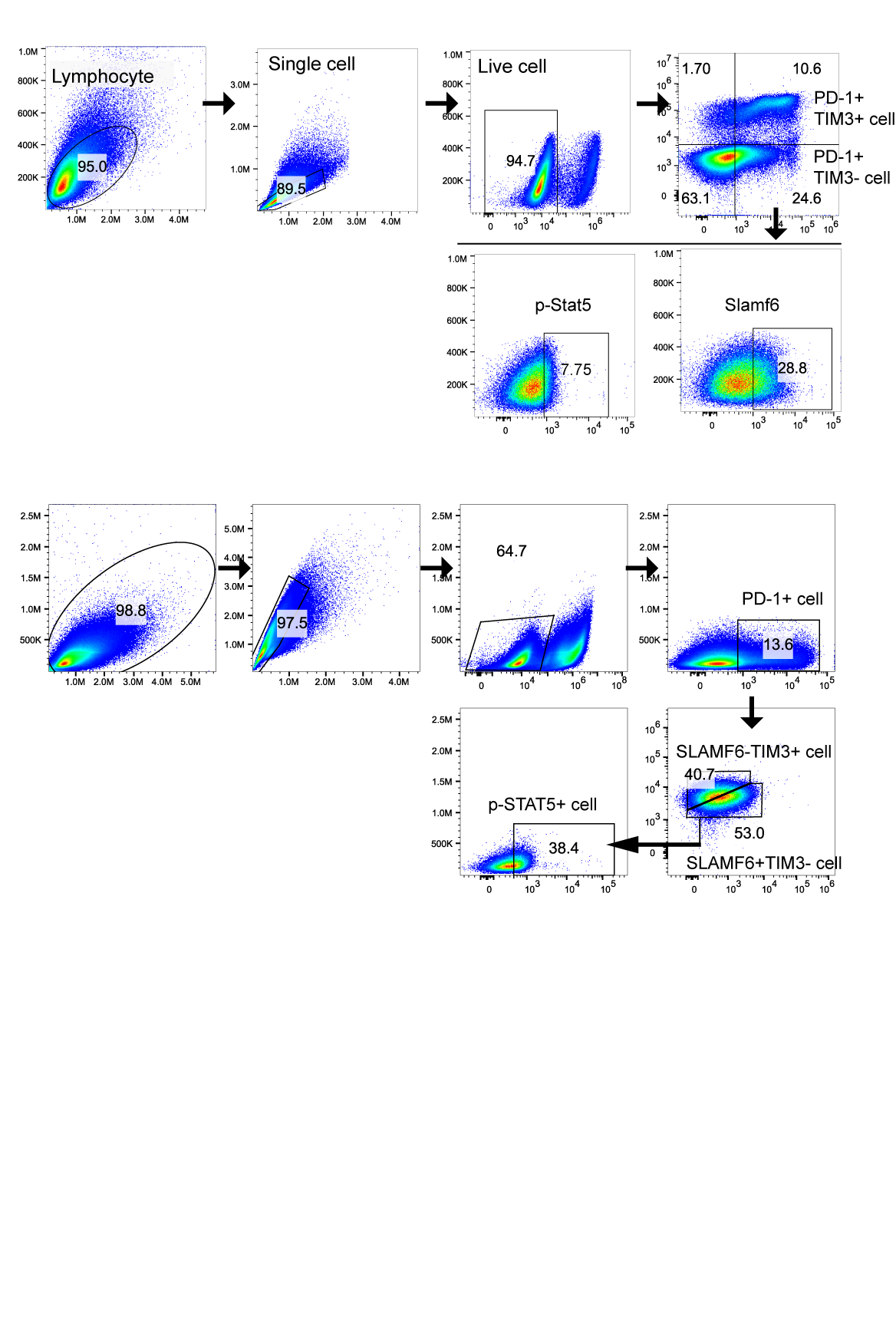


**Supplementary Fig. S13：**Gating strategies for the Sell(hi) neutrophils (CD45+CD11b+Ly6C-Ly6G+Sell+)


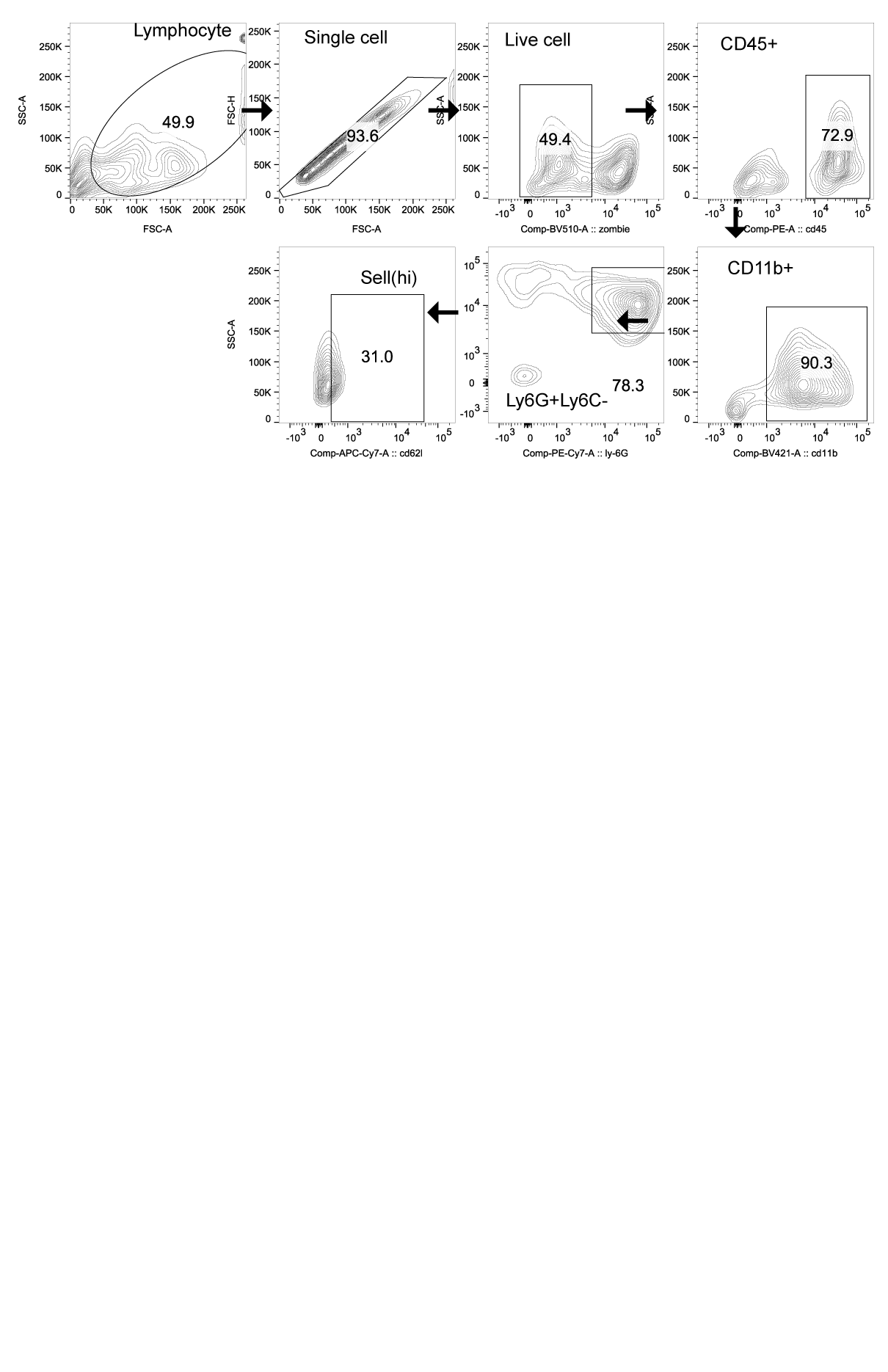

Supplement: Supplementary file 1 — Supporting Information [file ADVS-12-e10501-s009.docx]
